# The gene regulatory mechanisms shaping the heterogeneity of venom production in the Cape coral snake

**DOI:** 10.1186/s13059-025-03602-w

**Published:** 2025-05-19

**Authors:** Pedro G. Nachtigall, Brett R. Hamilton, Taline D. Kazandjian, Paolo Stincone, Daniel Petras, Nicholas R. Casewell, Eivind A. B. Undheim

**Affiliations:** 1 Centre for Ecological and Evolutionary Synthesis, Department of Biosciences, University of Oslo, PO Box 1066 Blindern, Oslo, 0316 Norway; 2 Centre for Microscopy and Microanalysis, University of Queensland, St Lucia, Brisbane, QLD 4072 Australia; 3 Centre for Snakebite Research & Interventions, Liverpool School of Tropical Medicine, Pembroke Place, Liverpool, L3 5QA UK; 4 Interfaculty Institute of Microbiology and Infection Medicine, University of Tübingen, Auf der Morgenstelle 28, Tübingen, 72076 Germany; 5 Department of Biochemistry, University of California Riverside, Riverside, 92507 CA USA

**Keywords:** Genomics, Mass spectrometry imaging, Gene regulatory network, Venomics, Elapidae, Toxin

## Abstract

**Background:**

Venoms and their associated glands and delivery structures have evolved numerous times among animals. Within these venom systems, the molecular, cellular, and morphological components interact and co-evolve to generate distinct, venom phenotypes that are increasingly recognized as models for studying adaptive evolution. However, toxins are often unevenly distributed across venom-producing tissues in patterns that are not necessarily adaptive but instead likely result from constraints associated with protein secretion.

**Results:**

We generate a high-quality draft genome of the Cape coral snake (*Aspidelaps lubricus*) and combine analyses of venom gland single-cell RNA-seq data with spatial venom gland in situ toxin distributions. Our results reveal that while different toxin families are produced by distinct populations of cells, toxin expression is fine-tuned by regulatory modules that result in further specialization of toxin production within each cell population. We also find that the evolution of regulatory elements closely mirrors the evolution of their associated toxin genes, resulting in spatial association of closely related and functionally similar toxins in the venom gland. While this compartmentalization is non-adaptive, the modularity of the underlying regulatory network likely facilitated the repeated evolution of defensive venom in spitting cobras.

**Conclusions:**

Our results provide new insight into the variability of toxin regulation across snakes, reveal the molecular mechanisms underlying the heterogeneous toxin production in snake venom glands, and provide an example of how constraints can result in non-adaptive character states that appear to be adaptive, which may nevertheless facilitate evolutionary innovation and novelty.

**Supplementary Information:**

The online version contains supplementary material available at 10.1186/s13059-025-03602-w.

## Background

Tissues and organs are composed of multiple cell types that vary spatiotemporally in their gene and protein expression profiles to create a final complex phenotype [1, 2]. This cellular heterogeneity is ubiquitously detected across different cell types [3, 4], but it is also often detected among similar cell types [5]. Heterogeneous expression among similar cell types can result from a differential intrinsic response to stress in the cellular environment, but also from activation of gene expression through the modular combination of distinct regulators, such as transcription factors (TFs) [6–9]. This heterogeneity among similar cell types appears to be ubiquitous in metazoans [10–13], suggesting that it may be an important player in the evolution of the body and cell biology. However, how this heterogeneous cell expression may impact the evolution of phenotypes can be difficult to decipher in highly polygenic traits.

One system that is well-suited to studying molecular underpinnings of phenotypic evolution is venom. Venoms have emerged independently in more than a hundred lineages across the animal tree of life and consist primarily of tens to hundreds of bioactive proteins and peptides, whose evolution can be studied individually [14]. These proteins and peptides (i.e., toxins) generally evolved via co-option and/or duplication of physiological genes followed by gene family expansions and functional diversification of paralogs [15–19]. However, the evolution of venom also requires the evolution of venom-producing tissues, attained either via the co-option of existing secretory glands or the development of new glands that contain specialized secretory cells adapted to rapidly produce large amounts of toxin. This combined set of molecular and cellular innovations is also associated with the co-option of regulatory modules that shape the final venom phenotype [20, 21]. By identifying the transcriptional regulatory elements associated with each toxin and comparing these to the evolution of toxin gene families, venoms provide an excellent opportunity to study the role that changes in regulatory pathways play in phenotypic evolution.

Interestingly, the distribution of toxins in venom-producing tissues tends to be highly heterogeneous [22–29]. In animals with a centralized venom system (i.e., all but cnidarians, which have venom-producing cnidocytes across all tissues), the often remarkable heterogeneity of toxins within venom glands can also include co-localization of secretory cells producing functionally similar secretions (e.g., [28, 30]). This distribution has been interpreted as an adaptation that enabled behavioral control over the composition of secreted venom [14]. However, there is emerging evidence that heterogeneous toxin distributions across glands are not necessarily adaptive, but instead reflect constraints on effective production of proteins by secretory cells [22, 28, 31, 32] that may provide an exaptation for subsequent evolution of behavioral control over venom secretion [33]. These findings suggest that cell-to-cell variation in gene expression plays a major role in the evolution of venoms and call for studies on how genetic regulatory networks (GRNs) can shape the cell-to-cell heterogeneity of gene expression of these polygenic and adaptive phenotypes.

Among venomous lineages, snakes are the most studied so far [34–36]. Recently, the spatial heterogeneous distribution and production of toxins in venom glands was identified by mass spectrometry imaging (MSI) of the venom glands from Elapidae and Viperidae, including both spitting (*Naja nigricollis*) and non-spitting cobras (*N. subfulva* and *N. haje*) [25, 27, 28] and the viper *Calloselasma rhodostoma* [28]. This heterogeneity has also been observed at the transcriptome level using single-cell RNA-seq (scRNA-seq) of venom gland tissue of the elapid *Aspidelaps lubricus* [37] and the viper *Crotalus viridis* [29]. While the first of these studies did not attempt to identify regulatory modules underlying the observed cellular heterogeneity [37], several regulons of toxin production were identified from *C. viridis* that may have evolved through co-option of TFs that interact with other biological pathways related to protein production and secretion [29]. However, a recent study on bulk transcriptome data comparing elapid and viper snakes suggest that their venom production is primarily controlled by distinct regulatory networks [38]. Thus, the regulatory mechanisms responsible for the distinct distributions of toxins in elapid venom glands and their associated evolutionary constraints remains unknown.

The Cape coral snake (*A. lubricus*) is a venomous snake species belonging to the Elapidae family. It is a relatively small snake, reaching up to 70 cm in length, has nocturnal and fossorial habits, and is mainly found in southwestern Africa [39]. It is a generalist predator that feeds primarily on amphibians, reptiles, and mammals [40], whose venom is mainly composed of a diverse set of three-finger toxins (3 FTx) with neurotoxic activity [41]. Recently, *A. lubricus* was used as a model system to develop venom-producing venom gland organoids that retained the heterogeneity of whole venom gland tissue [37]. Yet, although it represents a valuable resource for further functional studies aiming to understand the regulatory mechanisms involved in the generation of complex phenotypes, its genome sequence and regulatory elements involved in venom production remain unknown.

Here, we present a high-quality draft genome of the Cape coral snake (*A. lubricus*), that we leverage to gain locus-level resolution analyses of existing venom gland scRNA-seq data from the same species [37]. In addition to increased resolution of cell-cell differences in paralog expression, we identify several new, key regulatory elements underlying the specialization of toxin production by secretory cells. We then compare the evolution of toxins and their regulatory elements to the spatial distributions of toxins across the venom gland to provide new insight into mechanisms shaping the heterogeneity of venom production. Finally, we discuss the implications of our findings in understanding how cellular heterogeneity may impact phenotypic evolution.

## Results

### Genome assembly and annotation

The draft genome assembly returned a genome size of 1.82 Gb comprising 704 scaffolds with a N50 of 80.2 Mb and a L50 of eight (Additional file 1: Fig. S1). Genome completeness was evaluated using the tetrapod database (total of 5310 genes), revealing 94.3% complete and 1.5% fragmented BUSCO loci. Together, these metrics indicate that the genome assembly for *A. lubricus* is of high quality both in terms of contiguity and completeness. The repeat annotation revealed that 50.92% of the assembled genome consisted of repetitive sequences (Additional file 1: Fig. S2). These repeats accounted for 9.00% of tandem repeats and 38.78% of transposable elements (TEs). Among TEs, we identified 18.22% of long interspersed nuclear elements (LINEs), 6.02% of long terminal repeats (LTRs), and 11.08% of DNA transposons as the most abundant TE families. The high abundance of LINEs is in accordance with previous studies showing such a pattern for snakes [42–46].

The GALBA pipeline [47] returned 21,483 protein-coding genes, of which 20,350 (94.72%) had hits against the ENSEMBL database. Of these, TF prediction returned 1979 putative TF genes. Using ToxCodAn-Genome [48], we annotated 73 toxin genes from eighteen toxin families (Additional file 2: Table S1) comprising a set of known major and minor components of elapid venom [49]. Most of the toxin genes referring to major components in *A. lubricus* were represented by three-finger toxins (3 FTx), which comprised 27 genes, followed by snake venom metalloproteinases (SVMP), which comprised eight genes, by Kunitz-type toxin (KUN), which comprised seven genes, and by cysteine-rich secretory protein (CRISP), which comprised three genes. Among the 27 genes identified for the 3 FTx, three were most similar to cytotoxic-types (3 FTx-24, 3 FTx-25, and 3 FTx-26), whereas all other 3 FTx were most similar to neurotoxic types (Additional file 1: Fig. S3). Quantifying the gene expression in the bulk venom gland transcriptome showed that the major components are 3 FTx transcripts, comprising 69.14% of all toxin gene expression. These values are comparable to those of our reconstructed bulk venom gland transcriptome generated by summarizing counts from all cells in the scRNAseq data (hereafter referred to as pseudo-bulk), where 3 FTx accounted for 79.35% of all toxin gene expression (Additional file 1: Fig. S4). The expression pattern of 3 FTx is consistent with previous reports for the species [37, 41], which showed that its venom is mainly constituted of a diverse set of 3 FTx with neurotoxic activity [41]. Indeed, using a top-down proteomic approach—which is suitable for distinguishing peptide and small protein isoforms—we were able to confirm the presence of 21 of 27 3 FTx paralogs in the venom proteome, further supporting the agreement between the data obtained in both bulk and pseudo-bulk venom gland transcriptomes (Additional file 3). After merging toxin and non-toxin annotations, the final annotation set consisted of 73 toxin genes and 21,422 non-toxin genes with functional categorization (Additional file 2: Table S2).

### Single-cell venom gland expression profile

**Fig. 1:**
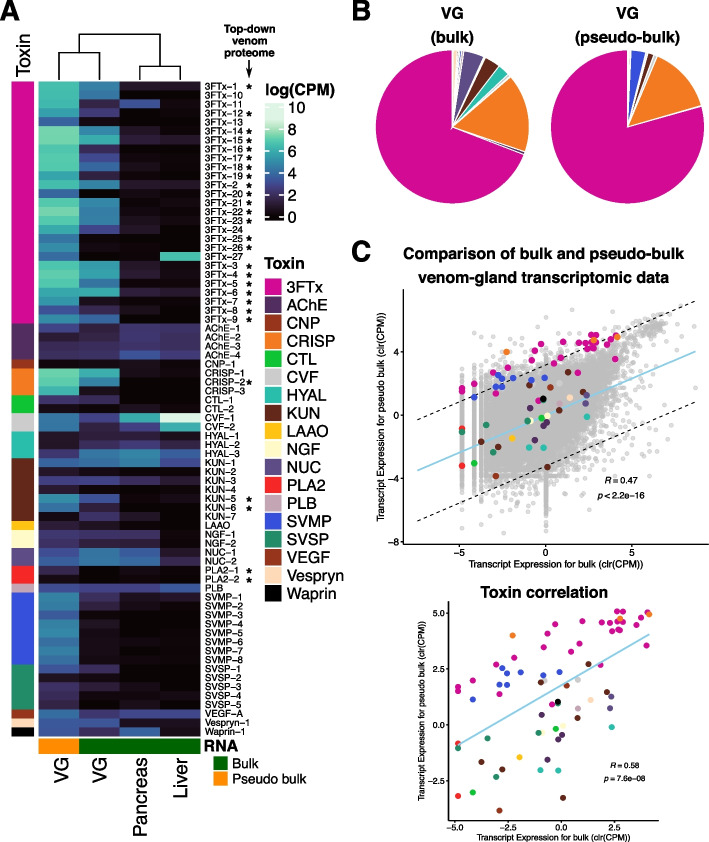
Comparison of venom gland scRNA-seq (accounted as pseudo-bulk) to bulk transcriptome data. **A** Heatmap of toxin gene expression in venom gland pseudo-bulk (in orange) and bulk RNA-seq of venom gland, pancreas, and liver (in green). Venom peptide and low molecular weight protein paralogs identified by top-down proteomics are marked with an asterisk. **B** Proportion of toxin expression in both bulk and pseudo-bulk venom glands. **C** Bulk and pseudo-bulk toxin expression profiles are correlated when comparing toxin and non-toxin genes as observed in the top scatter plot. The correlation is retained when analyzing only toxin genes. Dashed lines in the top scatter plots denote the 99% confidence interval of non-toxin expression and the light blue line shows the line of best fit based on orthogonal residuals. The light blue line in the bottom scatter plot denotes the line of best fit based on orthogonal residuals. The values within both scatter plots are the Pearson’s correlation coefficient (*R*) obtained when comparing the expression profile of both datasets. CPM, counts per million; 3 FTx, three-finger toxin; AChE, acetylcholinesterase; CNP, C-type natriuretic peptide; CRISP, cysteine-rich secretory protein; CTL, C-type lectin; CVF, cobra venom factor; HYAL, hyaluronidase; KUN, Kunitz-type toxin; LAAO, L-amino acid oxidase; NGF, nerve growth factor; NUC, nucleotidase; PDE, phosphodiesterase; PLA2, phospholipase A2; PLB, phospholipase B; SVMP, snake venom metalloproteinase; SVSP, snake venom serine protease; VEGF, vascular endothelial growth factor

To evaluate whether the scRNA-seq data reflected the overall expression profile of the whole venom gland, we compared the expression profile of the pseudo-bulk to the bulk RNA-seq of venom gland, pancreas, and liver (Fig. 1A, B). The direct comparison of whole tissue and pseudo-bulk expression data of the venom gland revealed no discrepancies between both types of data (Fig. 1C). This analysis revealed a consistent correlation between the scRNA-based pseudo-bulk and bulk venom gland, which confirms that the scRNA exhibits a similar expression pattern to the whole venom-gland tissue. Our data corroborates a previous report using data from a viper species that revealed a similar expression pattern between venom gland scRNA-seq and both bulk venom gland RNA-seq and venom proteome [29].

**Fig. 2:**
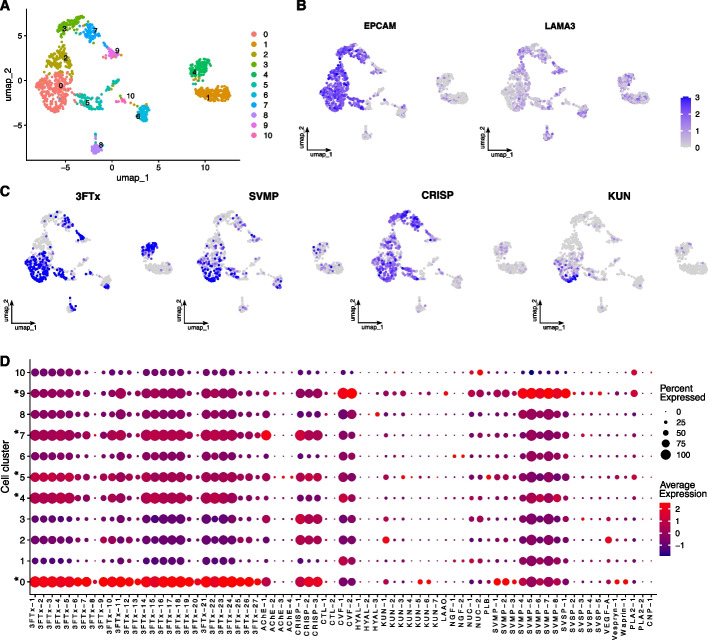
Cell clustering of the scRNA-seq data derived from the venom gland of *A. lubricus*. **A** Venom gland cell clustering (*n* = 1224) visualized using the UMAP approach. Colors represent each cell cluster (*n* = 11). **B** Expression levels of epithelial markers (i.e., EPCAM and LAMA3) in UMAP. Color represents a logarithmic scale of transcript expression with darker blue indicating higher expression level. **C** Average expression levels of the most abundant toxin families: 3 FTx, SVMP, CRISP, and KUN. **D** Expression profile of toxin genes in each cell cluster. The circle size represents the percent of cells within that cluster expressing that gene, whereas the colors represent the average expression of that toxin in that cluster (with red representing higher expression and dark purple representing lower expression). Asterisks (“*”) represent the toxin clusters

The scRNA analysis of *A. lubricus* yielded 1224 cells that were grouped into eleven naïve clusters based on their expression profile (Fig. 2A). The venom gland epithelial markers (i.e., LAMA3 and EPCAM; [37]) and the toxin expression profile across cells (Fig. 2B–D) allowed us to categorize these clusters into five clusters of toxin producing cells (clusters 0, 4, 5, 7, and 9; total of 629 cells) and six clusters of other cell types (clusters 1, 2, 3, 6, 8, and 10; total of 595 cells). Among the toxin clusters, clusters 0, 4, 5, 7, and 9 comprise 295, 110, 105, 75, and 44 cells, respectively. Clusters 0, 5, and 7 are mainly composed of 3 FTx and CRISP expression, of which cluster 0 presents the highest expression level of all 3 FTx paralogs. Cluster 4 presents mostly 3 FTx and SVMPs while cluster 9 presents high expression of SVMPs and cobra venom factors (CVFs). The clusters of other cell types (clusters 1, 2, 3, 6, 8, and 10) present low levels of toxin expression, which led us to classify cells within these clusters as non-toxin cells (Additional file 1: Fig. S5). Additionally, we performed subclustering of the 629 toxin cells to check whether they allowed us to capture any heterogeneity in toxin expression (Additional file 1: Fig. S6). This analysis resulted in seven distinct subclusters with heterogeneous toxin expression profiles, which indicates that the toxin producing cells are suitable for identifying modules of co-expression and characterizing genes regulating toxin expression.

### Modules of co-expression in toxin producing cells

**Fig. 3:**
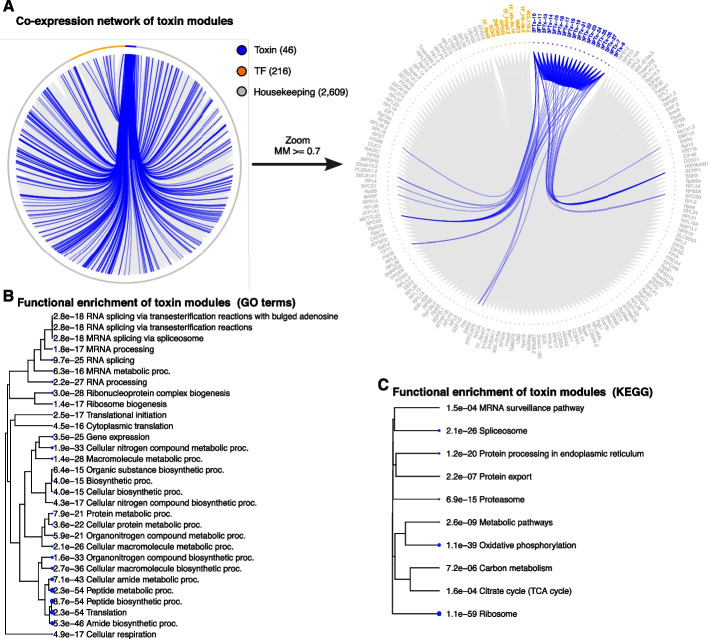
Modules of co-expression of toxin producing cells from the venom gland of *A. lubricus*. **A** The weighted gene co-expression network of toxin modules using toxin cells comprised 2871 genes. Of these, 46 were toxins (blue), 216 were transcription factors (orange), and 2609 were housekeeping (gray). On the left, a network with all genes within toxin modules. On the right, a zoom in showing genes filtered to have module membership (MM) greater or equal to 0.7 and adjacency greater than 0.01 for better visualization purposes. In both networks, the edges linking to toxin genes are highlighted in blue. **B** The 30 most significant GO terms of biological processes enriched in the toxin modules. **C** The 10 most significant KEGG pathways enriched in the toxin modules. Te pathways are shown based on their relationships of GO terms and the calculated *p*-values are shown before the GO names

We identified modules of co-expression following two approaches (see the “Methods” section for further details). First, we used all 1224 cells in the venom gland scRNA data (hereafter referred to as “all cells”), which consisted of cells classified as toxin- and non-toxin-producing cells by the clustering and subclustering steps of the scRNA analysis. Secondly, we used only the subset of cells comprising the toxin producing cells, which consisted of 629 cells with heterogeneous expression profiles of toxins as identified in the clustering and subclustering steps of the scRNA analysis (hereafter referred to as “toxin cells”). In the first analysis, the weighted gene co-expression network analysis (WGCNA) returned a total of 38 modules (Additional file 1: Fig. S7A), of which three modules comprised most toxin genes (i.e., 46 of 73 toxin genes). Among the toxin modules, one contained 37 toxins (27 3 FTx, eight SVMP, and two KUN) and 30 non-toxin genes (orange module in Additional file 1: Fig. S7A), one contained six toxins (three CRISP, and one of each Vespryn, AChE, and Waprin) and 3656 non-toxin genes (turquoise module in Additional file 1: Fig. S7A), while one contained three toxins (SVSP-3, VEGF-A, and KUN-1) and 1866 non-toxin genes (brown module in Additional file 1: Fig. S7A; Additional file 2: Table S3). The toxin modules in this strategy contained a total of 5598 genes, of which 46 were toxins, 449 were TFs, and 5103 were housekeeping genes (Additional file 1: Fig. S8A). In the second analysis, the WGCNA returned a total of 73 modules (Additional file 1: Fig. S9A), of which three modules comprised most toxin genes (i.e., 46 of 73 toxin genes). Among the toxin modules, one contained 33 toxins (26 3 FTx, four SVMP, two KUN, and one VEGF-A) and 41 non-toxin genes (mediumpurple3 module in Additional file 1: Fig. S9A), one contained 12 toxins (three CRISP, four SVMP, and one of each 3 FTx, KUN, Vespryn, AChE, and Waprin) and 2430 non-toxin genes (turquoise module in Additional file 1: Fig. S9A), while one contained one toxin (SVSP-3) and 354 non-toxin genes (blue module in Additional file 1: Fig. S9A; Additional file 2: Table S3). The toxin modules in this strategy contained a total of 2871 genes, of which 46 were toxins, 216 were TFs, and 2609 were housekeeping genes (Fig. 3A).

Interestingly, both strategies returned similar toxin genes within three highly correlated modules (Additional file 1: Figs. S7B, C, S9B, and C) presenting a positive and statistically significant correlation to the defined toxin cell traits (Additional file 1: Fig. S10). These 3 modules comprise the most highly expressed toxin genes in *A. lubricus* venom gland transcriptome, which also include the major components of the *A. lubricus* venom proteome [37, 41], and most of the non-toxin genes previously described to be part of the meta-venom network (e.g., MANF, TRAM1, PDIA6, PDIA3, and RPLP0; [20]). The functional enrichment analysis showed that genes within toxin modules (Fig. 3B, C; Additional file 1: Fig. S8B, C) are primarily related to transcription, translation, and protein export processes, which are the main biological processes for toxin production. We also noticed enriched gene-ontology (GO) terms related to protein folding, unfolding protein response, and response to endoplasmic reticulum stress, as previously observed ([20, 50, 51]; Additional file 2: Tables S4 and S5). In sum, our analyses revealed a set of co-expressed toxin and non-toxin genes consisting of putative regulators of toxin production in *A. lubricus*.

### Candidate TFs regulating toxin production

**Fig. 4:**
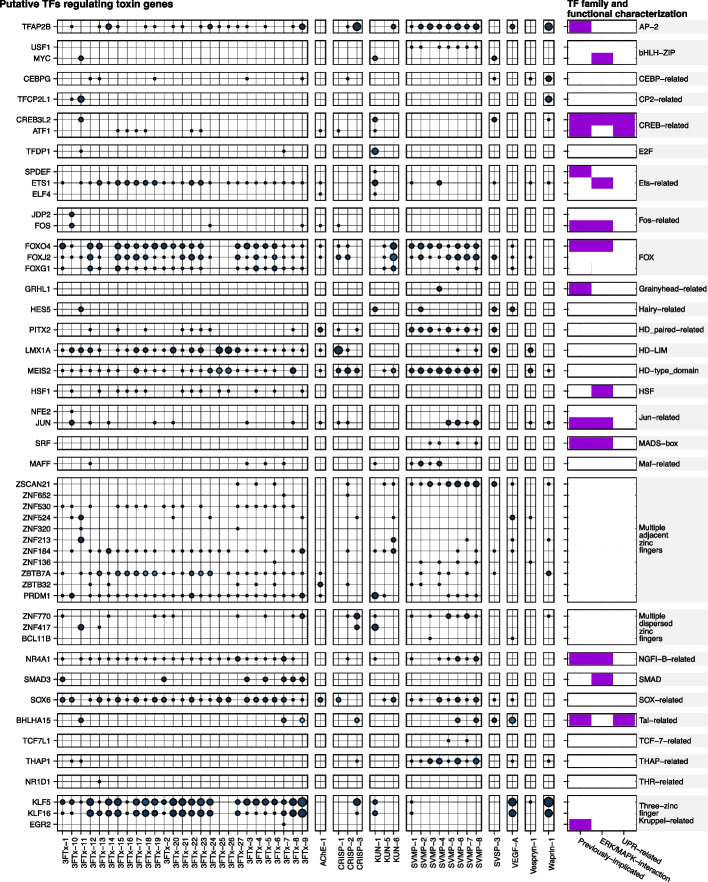
Transcription factors (TFs) identified as candidates to regulate the toxin gene expression using the toxin cells. Rows correspond to the TFs homologous to profiles at JASPAR in toxin modules and columns correspond to toxin genes in the toxin modules. Circles indicate transcription factor binding sites (TFBSs) in the promoter of the toxin gene. The size corresponds to the number of predicted TFBSs in a given promoter, in which larger circles represent more bound sites. The color corresponds to the network adjacency weights calculated using GENIE3, in which lighter colors represent higher weights. The columns on the far right show the family and function for each TF, indicating whether they were previously implicated in toxin production, directly interacting to the ERK/MAPK pathway, and/or interacting into the UPR pathway (purple squares)

It has previously been hypothesized that cellular constraints on protein secretion may drive toxin production heterogeneity within the venom gland [28]. To check if distinct genetic regulatory networks (GRNs) orchestrated by TFs could reflect the heterogeneity of venom production, we predicted binding sites for TFs within the toxin modules for both WGCNA strategies. We identified 449 and 216 TFs among the toxin modules using all cells and toxin cells, respectively. Then, we predicted the putative regulatory regions of toxin genes using computational approaches (see the “Methods” section for further details). Here, this set of putative, computationally-derived regulatory sequences based solely on physical distance are referred to as “promoters”. The transcription factor binding site (TFBS) prediction using the promoter of toxin genes integrated with the output from GENIE3 revealed 133 and 61 TFs as candidates to regulate toxin genes in *A. lubricus* using all cells and just toxin cells, respectively (Additional file 2: Table S6). The identified candidate TFs from all cells were homologous to 107 TFs in the JASPAR database and categorized into 42 TF families (Additional file 1: Fig. S11), while TFs identified from the toxin cells were homologous to 50 TFs from 28 TF families (Fig. 4), all of which except one (MEIS2) were also among the TFs identified from all cells. Several TFs were identified as being related to the extracellular signal-regulated kinase (ERK) and the unfolded protein response (UPR) pathways, which were previously shown to be evolutionary co-opted to regulate toxin production in snakes [20, 29, 50]. Additionally, we checked whether the candidate TFs had previously been shown to be implicated in venom production by surveying the literature for studies inferring putative regulators of toxin expression and production [38, 43, 50, 52–54].

Analyzing the four most abundant toxin families in the venom-gland transcriptome with the candidate TFs identified in the toxin cells, we detected 43, 30, 25, and 23 TFs binding into the promoter of 3 FTx, SVMP, KUN, and CRISP, respectively (see Additional file 2: Table S6 for full details). Among these TFs, we detected eleven TFs that were shared among all toxins, nine TFs that were specific to 3 FTx, five TFs specific to SVMP, three TFs specific to KUN, and no TFs that were specific to CRISP (Additional file 1: Fig. S12A; Additional file 2: Table S7). These patterns remained similar when identifying putative TFs using the candidate TFs from all cells (Additional file 1: Fig. S12A; Additional file 2: Table S8). Among the identified TFs, we noticed several TFs that participate canonically into the ERK and UPR signaling cascades. For example, CREB3L2 is a TF involved in ER stress and activator of the UPR [55] and has been described to be a modulator of toxin production in both vipers and elapids [29, 38]. However, there are also several TFs not directly linked into the ERK or UPR pathways, which indicates that modules from other pathways may also contribute to the cell-specific expression profile of toxin genes. The SVMPs presented a set of five specific TFs binding to their promoters, which included GRHL1, USF1, BCL11B, SRF, and TCF7L1. The GRHL1 was previously described to be a regulator of SVMPs in *Crotalus* species [29, 43, 50, 52], while TFAP2B was previously described to be implicated in the ontogenetic shift in the venom composition of *Crotalus adamanteus* [54].

While inspecting the 43 candidate TFs identified from the toxin cells that were predicted to bind to the promoter of 3 FTx paralogs, we noticed that a total of sixteen TFs are associated with the cytotoxic 3 FTx (i.e., 3 FTx-24, 3 FTx-25, and 3 FTx-26) and that these were all shared with neurotoxic 3 FTx (Additional file 1: Fig. S12B; Additional file 2: Table S7). In contrast, an additional 34 TFs were found to be specific to the neurotoxic 3 FTx paralogs. Among the sixteen cytotoxin-associated TFs, six are shared among all cytotoxins, while six are associated with only 3 FTx-24. The patterns were similar to the analysis performed using all cells (Additional file 1: Fig. S12B; Additional file 2: Table S8). Interestingly, phylogenetic analysis of the identified 3 FTx along with closely related elapid orthologs suggests 3 FTx-24 is basal to the other cytotoxins in *A. lubricus* (Additional file 1: Fig. S3). This relationship could explain the higher amount of shared TFs of this cytotoxin with its neurotoxic paralogs. Further supporting this hypothesis, the pairwise Jaccard similarity of TFs binding into 3 FTx revealed that 3 FTx-24 shares its TFs mainly with the neurotoxic 3 FTx-14 (Additional file 1: Fig. S13) and that this higher Jaccard similarity is mainly due to only two specific TFs shared between them when using only toxin cells (i.e., KLF5 and KLF16) and six when using all cells (i.e., CEBPA, KLF1, KLF5, KLF14, KLF16, and TFAP2 C). In sum, cytotoxins and neurotoxins are associated with a shared set of TFs, whereas the neurotoxic 3 FTx are also associated with an additional, specific, diverse set of TFs to regulate their expression. These findings indicate a more complex and fine-tuned regulatory mechanism in neurotoxins compared to cytotoxins.

### Promoter and coding sequence relationships of 3 FTx toxins

**Fig. 5:**
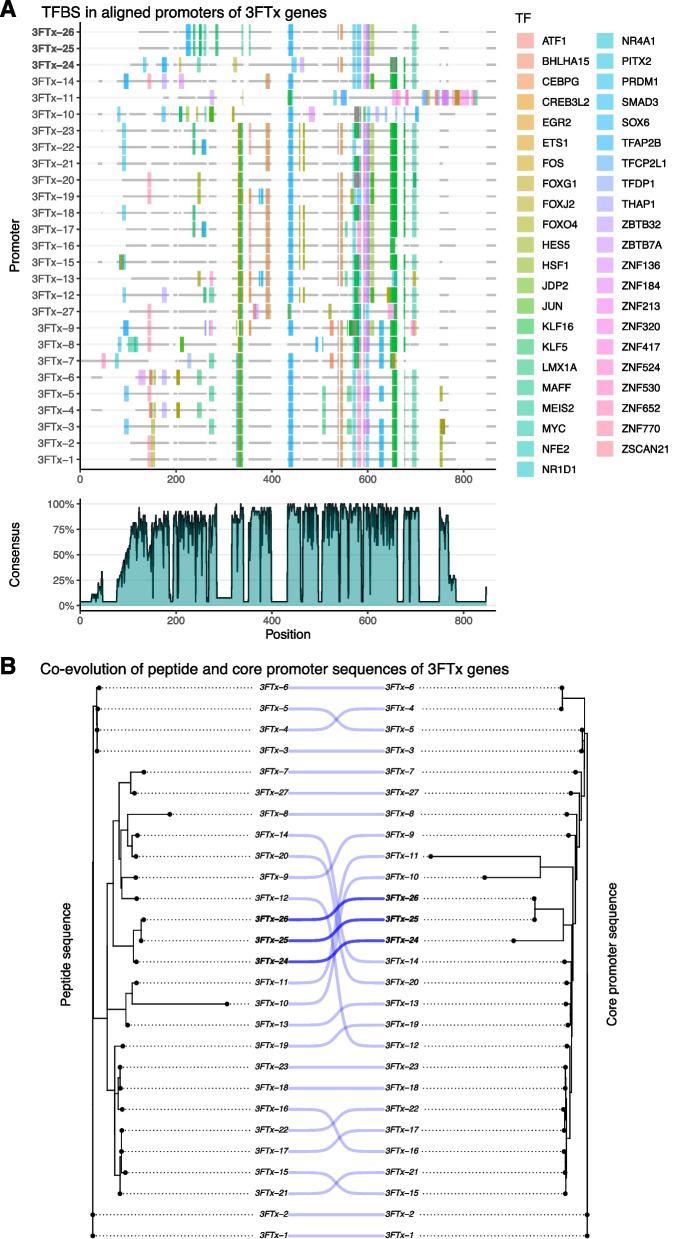
Transcription factor binding sites in promoter of 3 FTx genes. **A** Alignment and conservation of promoter sequences of 3 FTx with the TFBSs identified based on the toxin cells. The gray regions represent alignment gaps. The cytotoxins are highlighted in bold (i.e., 3 FTx-24, 3 FTx-25, and 3 FTx-26). **B** Co-evolution of peptide and promoter sequence of 3 FTX (see Additional file 1: Figs. S15 and S16 for bootstrap values; Additional file 1: Fig. S17 for correlation of patristic values). The phylogenetic trees inferred from both promoter and peptide sequences reveal similar evolutionary histories for both regions. The cytotoxins and their relationships are highlighted in bold

Given the shared set of TFs between neurotoxins and cytotoxins, and the set of neurotoxin-specific TFs, we next examined whether the evolutionary histories of 3 FTx genes could explain the similarities of their promoter regions. Aligning the promoter regions of all 3 FTx genes revealed a similar pattern of TFBSs among most neurotoxic 3 FTx, which differs from that observed for TFBSs among cytotoxic 3 FTx (Fig. 5A, Additional file 1: Fig. S14). To test whether these patterns could be due to phylogenetic relationships of the coding regions, we then inferred the phylogenetic relationships of 3 FTx paralogs using their peptide and promoter sequences and calculated their pairwise patristic distances (Additional file 2: Table S9). Comparing the topologies of the 3 FTx promoter and coding regions revealed a similar pattern of relationships of promoter and peptide sequences among 3 FTx types (Fig. 5B, Additional file 1: Figs. S15 and S16) as well as a positive correlation of patristic distances (*R* = 0.51, *p *< 0.001; Additional file 1: Fig. S17), which suggests a strong co-evolutionary relationship. These analyses also showed that the greater diversity in peptide structure among neurotoxic compared to cytotoxic 3 FTx is reflected in their promoters. Given the primarily neurotoxic venom of *A. lubricus*, and its generalist diet, the greater diversity of neurotoxic 3 FTx probably reflects a higher diversity of functional roles among paralogs in the venom. However, efficiently producing this neurotoxic arsenal also likely requires a greater degree of partitioning of paralog expression among secretory cells.

### The protein distribution of 3 FTx in the venom gland correlates to modules of TF

**Fig. 6:**
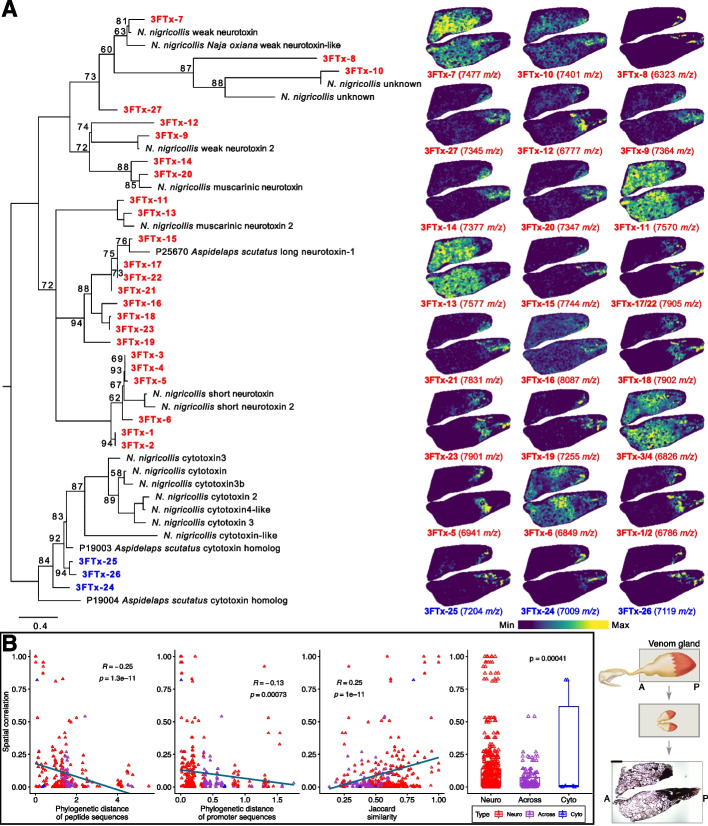
Spatial distributions of 3 FTx in the venom gland of *A. lubricus*. **A** On the left, the 3 FTx phylogeny with the bootstraps displayed at nodes and the cytotoxins and neurotoxins colored in blue and red, respectively. On the right, spatial distributions of 3 FTx as determined by MSI are shown as heat-maps across two near-serial sections from the same venom gland. Sections are positioned in mirrored orientation and heatmap color legend is shown below. Bottom right shows (from top to bottom) a schematic representation of the venom gland connected to the fang, the orientation of the sections used for the MSI, and the unstained sections used for MSI (bottom). The anterior region, which is near to the fang, is indicated with “A” and the posterior region, which is distant from the fang, is indicated with “P”. The scale bar represents a size of 500 μm. **B** Pairwise genetic distance of peptide and promoter sequences and Jaccard similarity of TFs correlated to the pairwise spatial correlations of the 3 FTx paralogs obtained in the MSI. Comparisons within neurotoxins, within cytotoxins, and across them are colored in red, blue, and purple, respectively

To investigate whether the 3 FTx heterogeneity observed in the scRNA-seq data were also observed at the protein-level in the venom gland of *A. lubricus*, we applied matrix-assisted laser desorption ionization (MALDI) mass spectrometry imaging (MSI). The resulting MSI spectra were dominated by strong signals in the region of mass-to-charge ratio (m/z) corresponding to masses typical of 3 FTx (Additional file 1: Fig. S18), which corroborates previous transcriptomic and proteomic findings that these are the main venom components [37, 41]. Matching the MSI peaks to known toxin masses and assigning functional activity through molecular phylogeny revealed that most 3 FTx are confined to distinct regions of the venom gland, which correlates to the heterogeneity observed at the transcriptomic level in the scRNA analysis (*R* = 0.24, *p*-value = 1.2e−10; Additional file 1: Fig. S19). In addition, we found that cytotoxins are confined to the posterior region of the venom gland, whereas the neurotoxic 3 FTx are distributed primarily in the anterior region of the venom gland (analysis of variance *p*-value = 4.1e-4; Fig. 6A). Interestingly, this is a similar pattern to those previously observed in spitting and non-spitting cobras of the genus *Naja* [25, 28]. We also detected a weak but significant negative correlation between 3 FTx paralog phylogenetic distances, of both peptide and promoter sequences, and their spatial correlation (Fig. 6B). Furthermore, we observed a strong and significant positive correlation when comparing the pairwise spatial correlation of 3 FTx to their respective overlap of TFs (i.e., Jaccard similarity; *R *= 0.25, *p*-value = 1e−11 using toxin cells shown in Fig. 6B; *R* = 0.3, *p*-value = 9.4e−16 based on all cells shown in Additional file 1: Fig. S20). We also confirmed that the Jaccard similarity positively correlates with the co-expression in scRNA data (*R *= 0.33, *p*-value < 2.2e−16 using toxin cells and *R *= 0.36, *p*-value < 2.2e−16 using all cells; Additional file 1: Fig. S21). These results indicate that modules of distinct TFs are likely to play major roles as regulators of the cellular heterogeneity observed in the *A. lubricus* venom gland.

**Fig. 7:**
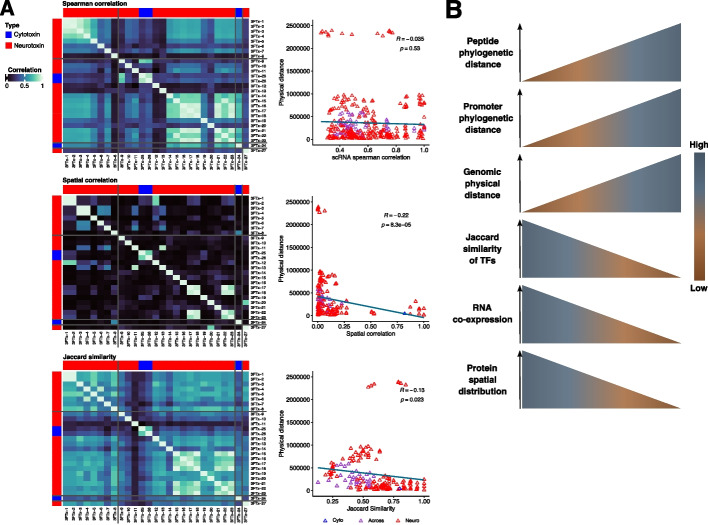
Correlation of spatial 3 FTx co-occurrence and their physical genomic distance. **A** Correlation of physical distance when compared to the following (from top to bottom): the spearman correlation of expression within cells in the scRNA-seq data, the spatial correlation of protein distribution within the venom gland, and the Jaccard similarity based on the toxin cells dataset. The heatmap shows each correlation and the dark gray lines indicate whether a 3 FTx is located within the same scaffold. The 3 FTx are sorted by their genomic distances. On the right, scatterplots show the correlation analysis. **B** Schematic overview of the analysis performed in the present study showing that modules of TF correlated to the heterogeneity of 3 FTx toxin production in *A. lubricus*

It has previously been shown that the heterogeneity of toxin gene expression in vipers could be a mechanistic consequence of the genomic context of toxin arrays (i.e., their intergenic distances) and the activity of distinct suites of TFs [29]. We therefore checked whether the genomic context of toxins could also be affecting the cellular heterogeneity observed in *A. lubricus* (Fig. 7). While we observe a stark mutually inverse co-expression pattern between adjacent paralogous loci of SVMP in *A. lubricus*, we did not observe this phenomenon among 3 FTx (Additional file 1: Fig. S22). Furthermore, the physical genomic distance of 3 FTx loci is not correlated with their co-expression in scRNA data (*R* = − 0.035, *p*-value = 0.53). In contrast, physical genomic distances of these loci are negatively correlated with both their Jaccard similarities (*R *= − 0.13, *p*-value = 0.023 based on toxin cells shown in Fig. 7A; *R* = − 0.21, *p*-value = 1e−4 based on all cells shown in Additional file 1: Fig. S23) and their spatial correlation in the venom gland (*R *= − 0.22, *p*-value = 8.3e−5; Fig. 7A). The physical genomic distance has a positive correlation to the phylogenetic distance of peptide and promoter sequences indicating that physical genomic distance reflects phylogenetic distance (Additional file 1: Fig. S24), whereas the phylogenetic distance of promoter and peptide sequences negatively correlates to the co-expression in scRNA data, spatial protein distribution in the venom gland, and Jaccard similarity of TFs (Additional file 1: Fig. S24). These results suggest that genes in close genomic proximity are more evolutionarily similar (in both promoter and peptide sequences) and more spatially correlated in the venom gland (in both transcriptomic and proteomic levels). Given the correlation between their Jaccard similarities and spatial correlations (Fig. 7B), the heterogeneity of 3 FTx observed in the venom gland of *A. lubricus* is likely primarily due to modules of TFs rather than physical genomic distance as previously observed in SVMPs of *C. viridis* [29].

**Fig. 8:**
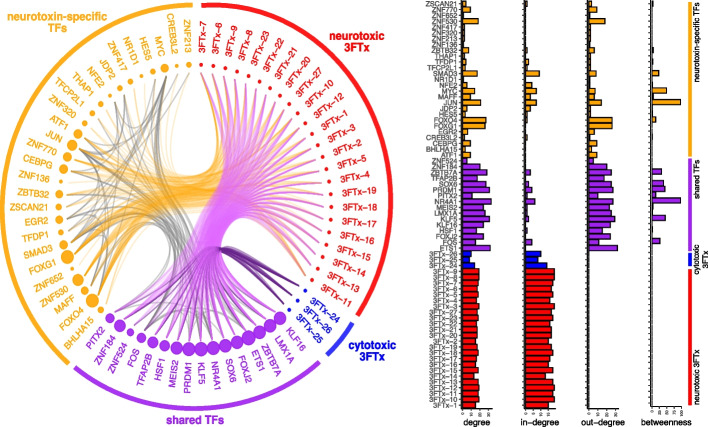
Modules of transcription factors in the genetic regulatory network (GRN) of 3 FTx. GRN inferred for the cytotoxic and neurotoxic 3 FTx using the toxin cells. Neurotoxins, cytotoxins, shared TFs, and neurotoxin-specific TFs are colored in red, blue, purple, and orange, respectively. The size of circles represents the out-degree of genes in the GRN. The orange edges indicate which neurotoxin-specific TF is binding to the neurotoxic 3 FTx. The magenta and dark purple edges indicate which shared TF is binding to neurotoxins or cytotoxins, respectively. The gray edges indicate the protein-protein interactions between TFs retrieved from the STRING database. On the right, the degree (total number of connections of each node), in-degree (number of incoming connections), out-degree (number of outgoing connections), and betweenness (number of times a node is the shortest path between other nodes) centrality measures obtained for each gene in the GRN showing relevant TFs controlling the 3 FTx expression profile observed

The gene regulatory network (GRN) of 3 FTx and their modules of TFs (Fig. 8; Additional file 1: Fig. S25) reveals an architecture containing a set of shared TFs and neurotoxin-specific TFs that can be responsible for the heterogeneity of toxin production in *A. lubricus*. The centrality measures calculated revealed the relevant roles played by specific TFs as modulators of the 3 FTx expression profile. Among the shared TFs, FOS, KLF5, NR4 A1, PITX2, PRDM1, SOX6, and ZBTB7 A have higher betweenness values, which indicates that these TFs are important regulators of both 3 FTx types. Among the neurotoxin-specific TFs, the TFs JUN, MYC, SMAD3, and FOXO4 have higher betweenness values, which suggests these TFs have greater influence on the modulation of neurotoxic 3 FTx expression. Thus, despite the large number of TFs detected to regulate the 3 FTx, a relatively small set of TFs appear to be of high relevance in the regulatory architecture of 3 FTx, suggesting they are major regulators of 3 FTx expression. In contrast, most TFs detected had lower relevance in the GRN, which may indicate a recent co-option into the GRN by targeting specific 3 FTx and not having stronger interactions with other TFs.

## Discussion

A wide diversity of cell types with distinct expression profiles are widely observed throughout the evolutionary history of species [56]. Several studies have provided new insights into how this cell type diversity exists both within and across organs such as central nervous systems, reproductive tissues, and immune systems [57–60]. While heterogeneous expression profiles of cells can be a result of differential response to tissue stress or differential transcriptional states of cells, different progenitors during development also lead to stable populations of differentiated cells within tissues [61]. Recently, this cellular heterogeneity was described in salivary glands of vertebrates [62] and venom glands of venomous snakes [28, 29, 37]. Leveraging a high-quality genome assembly, our findings provide additional insight into the remarkable cellular heterogeneity of gene expression in snakes via the venom gland of *A. lubricus*. Supporting the distinctiveness of these toxin-producing cell subtypes, our integrative analysis revealed that the heterogeneity observed in the scRNA-seq is also observed at the proteomic level within the venom gland tissue and that this distribution correlates to similar modules of TFs. Taken together, we hypothesize that a cellular diversity governed by the developmental fate of cell populations led to the heterogeneous location of 3 FTx within the venom gland, which is intrinsically controlled by specific modules of TFs.

Interestingly, the heterogeneous distribution of 3 FTx observed in *A. lubricus* has also been observed in other closely related elapids [25, 27, 28]. These studies revealed similar localization of 3 FTx types in the venom gland, with cytotoxins generally located in the posterior region and neurotoxins predominantly located in the anterior region. Our results suggest that these toxin distributions are likely controlled by associated molecular machinery mechanisms, such as the modules of TFs regulating the 3 FTx, that are conserved across closely related toxin orthologs belonging to the same toxin sub-family. These findings also support the hypothesis that the spatial segregation of functionally distinct toxins is not an adaptive feature related to venom function [22, 28]. Instead, the differentiation of toxin-secreting cell subtypes that result in these distinct distributions of toxins in the venom glands of snakes support the hypothesis that this segregation reflects constraints related to toxin production. Venom plays a key ecological role in most venomous snakes and substantial metabolic investment is usually made to ensure rapid repletion of spent venom [63]. Subdividing the production of toxins among populations of secretory cells could expedite this process by limiting the number of toxin-associated components (i.e., co-factors) that are expressed by each cell.

The subdivision of toxins among populations of secretory cells in the venom gland has some potentially important implications for the evolution of toxins in that the promoter and toxin-coding regions of toxin paralogs would likely be under selective constraints at different levels. Toxin paralogs typically evolve by selection-driven functional diversification in response to antagonistic co-evolutionary relationships between their overall venom phenotype (the sum of all toxins) and the molecular targets of their prey [64, 65]. While the promoter regions of these paralogs are also affected by selection on the overall venom phenotype through the quantitative contributions of their associated toxin-coding region [66], we speculate that the observed heterogeneity in toxin production suggests promoter regions are under additional selection to facilitate and maintain cell- or cell-population-specific expression within the venom gland. The current dataset available does not allow us to perform a proper selection analysis and test if the pattern observed has been fixed by selection, genetic drift, or a combination of both processes. However, this presents an interesting arena of intra-gene evolutionary conflict to investigate in future studies.

While the distribution of distinct toxins across the venom gland is not adaptive from a functional perspective, the distinct regulatory modules that underlie this differentiation could facilitate adaptation through rapid phenotypic shifts in venom function. Cytotoxic venoms may have evolved primarily as a defensive innovation in Elapidae and have co-evolved with hooding behaviors on two independent occasions [67]. Further, the evolution of explicit defensive use of venom by “spitting” has evolved on three separate occasions in Elapidae, each time associated with abundant use of cytotoxic 3 FTx paralogs alongside upregulation of venom phospholipase A2 [68]. Upregulation of cytotoxic 3 FTx has also been proposed to be a mechanism for the evolution of defensive venom use in several non-spitting elapid snakes [67]. In contrast, a secondary loss of cytotoxicity with a reduction of defensive behaviors occurred in some elapids, which possesses a neurotoxic venom with high abundance of neurotoxic 3 FTx paralogs [67]. The modularity of the GRN underlying the expression of neurotoxic versus cytotoxic 3 FTx is likely to have enabled this repeated, convergent phenotypic shift through reduced pleiotropic constraints between groups of neurotoxic and cytotoxic paralogs. As such, the potential adaptation to alleviate constraints on toxin production that these GRN modules represent, may also have been exapted to enable repeated functional innovation in elapid snakes, similar to what has been observed in the venomous giant centipedes [33].

Previous studies have identified the UPR and ERK pathways as key components in the production as well as the evolution of venom in snakes [20, 21, 38, 50]. These pathways are also important for the regulation of toxin expression in *A. lubricus*, with two and ten of the 28 TF families identified as being associated with toxin expression are known to integrate the UPR and ERK pathways, respectively. However, our findings also provide both additional and new insights into the TFs implicated in expression of the primary venom components of elapids. These insights include the identification of three ERK-associated TF families not previously implicated in the expression of snake venom toxin genes, as well as identification of regulatory pathways that have to our knowledge not previously shown to be associated with regulation of snake toxin genes. Among these TFs were BHLHA15, which is related to the maintenance of secretory cell architecture [69], and PRDM1 and SOX6, which are related to maintaining muscle cell architecture [70, 71]. Taken together, these findings suggest the evolution, and perhaps emergence, of novel traits can be associated with the recruitment of regulatory elements from multiple and distinct pathways.

While our findings both show similarities in high-level pathways and identify “new” venom-associated regulatory elements of snake venoms, they also highlight differences in toxin gene regulation that exist in venomous snakes, even within the same taxonomic family. For example, we found no evidence that physical distance between paralog copies in a tandem array may affect the regulation of 3 FTx paralog expression, which was previously observed in SVMP paralogs in rattlesnakes [29]. While we observed a stark mutually inverse co-expression pattern between adjacent paralogous loci of SVMP in *A. lubricus*, we found several examples of adjacent 3 FTx paralogs showing a high degree of co-expression, most likely due to closely related coding and promoter regions. These findings suggest that the regulation of 3 FTx paralogs in *A. lubricus* is primarily driven by TF-mediated regulation, perhaps due to the smaller physical size of the loci in this toxin family. While our observations are similar to those recently reported for another elapid species [38], there are also striking differences between putatively important TFs in *A. lubricus* and *Pseudonaja textilis*. For example, Modahl et al. [38] identified specificity protein 1 (SP1), forkhead box N2 (FOXN2), and ligand-dependent corepressor (LCOR) as the most highly upregulated TFs in response to depletion of the venom gland. In contrast, we found no evidence that these TFs regulate the expression of toxins in *A. lubricus*. However, we did identify an isoform of cAMP-responsive element binding protein 3-like (CREB3L3; CREBL3L2 in *A. lubricus*), which has been identified as a TF central to venom production in the viperid *C. viridis* but not the elapid *P. textilis*. While we note that the toxin-associated TFs identified in both elapid species is solely based on in silico analyses, this apparent lack of taxonomic signal in toxin-associated GRN, even within homologous toxin families, is striking.

In addition to differences in apparent key TFs for the expression of toxins in *A. lubricus* compared to other venomous snakes, alignment of the promoter region of all 3 FTx genes revealed that no single predicted TF binding site was conserved across the promoter regions of all venom 3 FTx paralogs (Fig. 5A). Instead, regulation of 3 FTx—﻿and hence also to some degree secretory cell sub-specialization—﻿appears to be a result of unique combinations of TF binding sites. We also observed similar variation in predicted TF binding sites between different paralogs in three other multi-copy toxin gene families. However, unlike 3 FTx, these families shared one (KUN: ZNF184; CRISP: MEIS2) or seven (SVMP: MEIS2, THAP1, FOXO4, FOXJ2, PITX2, TFAP2B, ZSCAN21) predicted TF binding sites among the promoter regions of all paralogs. While the differences in the degree of regulatory conservation of paralogs among toxin families may reflect either gene family size, structural diversity, or both, these findings suggest that barcode-like patterns, as opposed to distinct TFs, form the primary regulators of the expression of distinct toxins. Indeed, the repertoire of TF binding sites has been shown to be more relevant than the regulatory sequence itself in maintaining cell type-specific regulatory networks across broad evolutionary scales of metazoans [72], while similar barcode-like patterns has been observed across cell lines during embryogenesis in animals [73, 74].

While all paralogs are regulated by at least three components of the ERK-pathway, the lack of universally conserved regulatory elements among 3 FTx toxin paralogs in *A. lubricus* is striking. Given the strong correlation between coding and promoter phylogenetic relationships, we hypothesize that the gene regulatory elements of venom production may evolve rapidly to facilitate—﻿and accommodate—﻿increased expression levels of specific toxins [75], for example after events of functional innovation of toxin paralogs. We further hypothesize that these rapid changes in gene expression are primarily enabled by changes in the regulatory “barcode” through loss and/or gain in TF binding sites. While these hypotheses remain to be tested, they would explain the large differences in regulatory elements associated with toxin expression observed between *A. lubricus* and *P. textilis* [38]. They also provide a mechanism for how gene regulatory networks may facilitate rapid phenotypic shifts that can be observed among adaptive phenotypes such as venom.

In addition to insights into the regulatory network of venom production in *A. lubricus* and its conservation across elapid and viperid snakes, our findings raise questions about the role and evolutionary conservation of toxin regulatory elements. Future studies should therefore examine whether the modular regulatory architecture of toxin expression is conserved or highly variable on a narrow and/or a broad scale, as well as experimentally test the contribution of specific TFs to toxin production. Our results provide an important foundation for such experimental functional genetics studies, facilitating the use of venom gland organoids and gene editing techniques to confirm the activity of specific TFs in regulating the expression of toxin genes. To test the generality of such evolutionary strategies for regulating venom and for understanding the proximate and ultimate causes, consequences, and origins of cellular heterogeneity, further studies must generate single-cell multiomics data (i.e., per-cell ATACseq coupled with RNA sequencing) of several venomous snake species for a comprehensive comparative analysis. In this sense, the combination of high-resolution molecular technologies with validation experiments using organoids and a comprehensive comparative analysis will help to provide deeper insights into the biology of venom glands and the evolutionary dynamics of venom production. Understanding these processes holds potential applications in biotechnology, medicine, and evolutionary biology, offering new avenues for research and innovation.

## Conclusions

Our analysis revealed a high degree of specialization and sub-specialization of toxin production among venom gland secretory cells in *A. lubricus*, which, together with previous findings, suggests that cellular heterogeneity in gene expression within the venom gland is a fundamental aspect of venom production. Such compartmentalization of toxin production is strictly regulated by combinations of TFs and TF binding sites that closely reflect toxin phylogenetic relationships and explain spatial clustering of functionally similar toxins. At the same time, the individual elements of these regulatory barcodes are highly variable, both among toxin paralogs and between orthologous toxin gene families. This variability in regulatory element composition likely facilitates the rapid phenotypic evolution commonly observed in venoms of snakes, such as the repeated evolution of defensive venom in elapid snakes. An interesting direction of future work would be to test whether this rapid compositional evolution of regulatory elements could provide a mechanism of facilitating phenotypic shifts in other venoms, and adaptive polygenic traits in general.

## Methods

### Genome sequencing and assembly

To sequence the genome of *A. lubricus*, we extracted high molecular weight DNA from 200 μL of blood using the standard MagAttract HMW DNA protocol (Qiagen, Germany). The blood sample was collected as part of routine veterinary care of a captive female specimen housed and maintained at Leiden Zoo, the Netherlands. DNA integrity was assessed using a Fragment Analyzer (Agilent, USA). DNA was fragmented to 15–20 kb fragments using Megaruptor 3 (Hologic, USA) before the library was prepared using Pacific Biosciences protocol for HiFi library prep using SMRTbell^®^ ExpressTemplate Prep Kit 2.0. The resulting library was size selected with a 10 kb cut-off using BluePippin (Sage Science, USA) and sequenced with two 8M SMRT cells on a Sequell II instrument (Pacific Biosciences, USA) using Sequel II Binding kit 2.2 and Sequencing chemistry version 2.0. Loading was performed by adaptive loading, using a movie time of 30 hours with a 2-h pre-extension time, yielding a total of 11,818,443 reads with average polymerase read length of 85–86 kb and a total polymerase bases of 1016.2 Gb. Circular consensus sequences (CCS) were generated using the CCS pipeline (SMRT Link version 10.2.0.133434), resulting in 495,861 HiFi reads (> Q20) with a mean length of 16 kb, median quality Q32–Q33, and a totalling 65.99 Gb. Cutadapt version 4.4 [76] was used to remove remaining adapter sequences. The trimmed HiFi reads were assembled using hifiasm version 0.15.1-r329 [77] with default settings. The resulting genome assembly contiguity was calculated using Quast version 5.2.0 [78] while completeness was assessed by comparing against universally conserved single-copy orthologs from Tetrapoda (tetrapoda_odb10) using BUSCO version 5.0.0 [79]. Assembly statistics were summarized and visualized using BlobTools version 1.1 [80]. The genome assembly is deposited in NCBI under the accession number JAOANS000000000 [81].

### Genome annotation

We annotated repetitive regions and transposable elements (TEs) using RepeatModeler2 and RepeatMasker as previously described [82]. We used the RepeatModeler2 version 2.0.1 [83] to generate a de novo species-specific repetitive-sequence and TE library. We split the library into “known” and “unknown” sets as output by RepeatModeler2. The “unknown” set was classified using DeepTE version 1.0 [84] with the model designed for metazoans. To remove false-positive repetitive elements, we filtered out any sequence classified as “NonTE” using TERL version 1.0 [85]. Then, the species-specific TE library (i.e., the “known” set and the “unknown” re-classified set) was merged to a curated TE library available for snakes [42] to generate a final TE library, which was used to perform the repetitive annotation using RepeatMasker version 4.1.1 (https://www.repeatmasker.org/). The divergence between the individual TE copies versus their consensus sequences based on CpG-adjusted Kimura distance was estimated using built-in scripts from RepeatMasker.

Gene annotation was performed using the soft-masked genome and the GALBA pipeline version 1.0.11 [47]. We used the proteins annotated in the *Naja naja* genome available at Ensembl database (release 112) as the protein source for GALBA annotation. To check for the quality of predictions, we BLAST search the predicted proteins against the annotations available for mouse, chicken, green anole, central bearded dragon, komodo dragon, common wall lizard, mainland tiger snake, and eastern brown snake available in the ENSEMBL database (release 112). Additionally, we assigned gene names and functional annotations for the genes predicted by GALBA through orthology using the complete set of annotations available for *Naja naja*, *Gallus gallus*, and *Mus musculus* in the Ensembl database (release 112), and also the annotations available for the recently published genome of *Crotalus adamanteus* [54]. We used the peptide sequences as input to assign orthology using OrthoFinder [86], which allows us to infer the biological roles and pathways of the predicted proteins in *A. lubricus* genome. To identify genes potentially coding for transcription factors, we scanned the peptide sequences of genes using DeepTFactor [87], which is a high-throughput deep-learning sequence-based approach to identify transcription factor potential of protein-coding genes and it has been shown to perform a reliable prediction of transcription factor candidates in snakes [54].

To annotate toxins, we used ToxCodAn-Genome version 1.0 [48] with default parameters and followed their guide to ensure a confident toxin annotation set. Briefly, the bulk venom-gland transcriptomic data was assembled and annotated using ToxCodAn version 1.0 [88] with default parameters to generate a species-specific toxin database. The species-specific and the Elapidae toxin databases were used as database sources to annotate the toxins in the genome using ToxCodAn-Genome version 1.0 [48]. We then generated a final annotation set by merging the toxin and non-toxin annotations, which consisted in removing genes overlapping the annotated toxins from the GALBA annotation set to avoid missannotations in the final set.

To characterize the 3 FTx functionally as neurotoxins or cytotoxins, we performed an homology analysis using the peptide sequences of 3 FTx from *A. lubricus* to the known sequences available for the closely related species *A. scutatus* (P19003, P19004, and P25670 from Uniprot) and *N. nigricollis* [28]. Specifically, the mature peptide sequences (i.e., with no signal peptide) were aligned using MAFFT version 7.450 [89] and the phylogenetic tree was inferred using IQ-TREE version 1.6.12 [90] with the following parameters “-m TEST -b 1000 -alrt 1000”. The relationships allowed us to infer which 3 FTx from *A. lubricus* are neurotoxic or cytotoxic representatives.

### Single-cell RNA-seq data analysis

We used the single-cell RNA-seq data (scRNA) generated for the venom gland tissue as previously described (PRJNA531889; [37, 91]). The scRNA reads were demultiplexed based on their barcode and UMI sequences using the SingleCellMultiomics approach developed at the Van Oudenaarden lab (https://github.com/BuysDB/SingleCellMultiOmics). Then, the demultiplexed reads were mapped using STAR version 2.7.11 [92]. As reference for mapping reads, we used the extended gene annotation, which comprises the entire gene (i.e., coding sequences and introns) with 200 bp upstream and 500 bp downstream, and removed mitochondrial and ribosomal genes. Cell assignment and gene counts were performed using the scanpy package [93] by filtering cells with at least 1000 counts and at least 100 different genes to be expressed, which returned a final set with 1224 cells to be used in downstream analysis. The cell clustering was performed using the Seurat package [94], which included normalization, clustering and subclustering, dimensionality reduction, co-expression correlation, and plots of feature expression. To characterize cell clusters containing toxin producing cells, we analyzed the expression of two epithelial markers (i.e., LAMA3 and EPCAM), which were previously shown to be markers for toxin production cells [37], and the expression profile of toxin genes among clusters. Cells within the toxin production clusters were used as input for detecting modules of co-expression.

To check if the venom gland scRNA obtained a similar heterogeneity to a bulk venom gland RNA-seq data, we compared the expression profile of the venom gland scRNA to that obtained for bulk venom gland, pancreas, and liver (PRJNA531889; [37, 91]). First, we mapped the bulk RNA-seq data in the genome using STAR version 2.7.11 [92]. We retrieved the gene expression counts using featureCounts version 1.6.3 [95], and incorporated the venom gland scRNA as a pseudo-bulk by summoning the counts obtained in all cells. Then, the count table was imported and normalized using the trimmed mean of *M*-values in edgeR package [96]. To compare the expression profiles of the pseudo-bulk and bulk venom gland, we calculated a pairwise null distribution of expression divergence based on non-toxin expression levels [97]. The data was centered log-ratio (clr) transformed to normalize the expression distributions while accounting for the compositional nature of the relative expression values (i.e., CPM). Genes highly divergent in expression level (when comparing the pseudo-bulk and bulk venom-gland) may present a divergence outside the 99 th percentile of the centered log-ratio transformed distribution of non-toxins.

### Modules of co-expression within toxin cells

We performed a weighted gene co-expression analysis (WGCNA) to find regulatory elements shaping the heterogeneity of toxin production. To do this, we performed this analysis following two strategies: (1) using the counts of the 1224 venom-gland cells in the scRNA-seq dataset (“all cells”); and (2) using the counts from the 629 toxin cells (“toxin cells”). We opted to apply both strategies because there is no benchmarking analysis showing which is the best strategy, but some studies argue that using a subset of cells helps to refine the co-expression modules identified within the cells analyzed because correlation network approaches, like WGCNA, are sensitive to data sparsity [98–101]. In both strategies, the raw counts were normalized using the Trimmed Mean of M-values in edgeR package [96]. Then, the weighted gene co-expression analysis was conducted with the normalized data using the WGCNA package [102]. We set a soft threshold based on outputs of the “pickSoftThreshold” function from the WGCNA package to attain scale-free topology. A minimum module size of 30 and a correlation threshold of 0.2 were used to merge modules with similar expression profiles. In both strategies, the most abundant and major components of *A. lubricus* venom were detected among 3 highly correlated modules, which were considered the toxin modules in downstream analysis. The network of co-expression was plotted using the igraph package [103]. The toxin modules were analyzed to check for TFs co-expressed with toxin genes, which indicates their relationships to toxin production and were further analyzed for prediction of binding sites. We also analyzed the active biological processes active in the toxin modules. The GO term and KEGG pathway enrichment analysis was performed using the ShinyGO package version 0.8 [104] by setting the genes in the toxin modules as the test data and all other genes as the background. We set the false discovery rate cutoff to 0.05 and minimum pathway size to 15, but set the number of pathways to show to 30 and 10 for GO term and KEGG pathway, respectively. Additionally, we ran the GOstats package [105] to have a full list of GO terms within the toxin modules. For this analysis, we set the genes within toxin modules as the test data and all genes as the “universe.”

### Prediction of TFBS and GRN architecture

To identify the transcription factor binding sites (TFBS) for TFs within the venom network in promoter of toxin genes, we first identified candidate transcription start site (TSS) for each toxin gene by combining TSS prediction using TSSFinder [106], by setting it to use the pre-built model available for chicken, and searching the genome for the 5’UTRs identified using UTRan [53] with the toxin transcripts and the venom-gland transcriptome data used in the genome annotation step. The TSS was manually reviewed by considering the overlap of TSS prediction and the best matching 5′UTR, which was screened using BLAST search setting 100% coverage and 95% percent identity. Then, 500 bp upstream the TSS was used as the promoter for TFBS screening.

The TFBS prediction was conducted using CiiiDER version 0.9 [107] with the non-redundant vertebrates set from JASPAR 2024 database as source. TFs containing at least one binding site in the promoter of toxin genes were kept as candidates for regulating the toxin expression profile. We also calculated the TF-gene network adjacency weights using the random forest regression algorithm from the GENIE3 package [108]. Additionally, we screened the literature to check if the candidate TFs were previously implicated as regulators in toxin production [38, 43, 50, 52, 53] and if they play roles in the extracellular signal-regulated kinase (ERK) and the unfolded protein response (UPR) pathways, which were previously shown to be relevant biological pathways in toxin production of snakes [20, 50].

To complement the TF-gene network, we integrated the protein-protein interactions among the candidate TFs using the STRING database (accessed in November 2024; [109]). We also calculated four centrality measures for the genes within the network to identify relevant TFs regulating the toxin production: (1) the degree, which reveals the total number of connections of each node (i.e., higher numbers means the gene is interacting, regulating and/or being regulated by more genes); (2) the in-degree, which reveals the number of incoming connections (i.e., higher values mean the gene is interacting with or being regulated by more genes); (3) the out-degree, which reveals the number of outgoing connections (i.e., higher values mean the gene is interacting with or regulating more genes); and (4) the betweenness, which reveals how often a node is the shortest path between other nodes in the network (i.e., higher values mean the gene can be a key gene in a regulatory module).

To investigate conservation and differences in TFBSs among promoters, we aligned the promoter sequences and TFBS positions using MAFFT version 7.450 [89] and plotted them using the ggplot2 package. We aligned the peptide sequences of toxin paralogs using MAFFT and generated a phylogenetic tree for both peptides and promoter sequences to investigate whether their relationships were similar using IQ-TREE version 1.6.12 [90] with the following parameters “-m TEST -b 1000 -alrt 1000” and plotting both trees using the phytools package. We used the phylogenetic trees to calculate the pairwise phylogenetic distance (i.e., pairwise patristic distance) of peptide and promoter sequences across 3 FTx paralogs.

To investigate shared and specific TFs regulating the toxin genes, we analyzed the set of TFs overlapping between the main toxin families (i.e., 3 FTx, SVMP, KUN, and CRISP) and also among the 3 FTx types (i.e., cytotoxins and neurotoxins). For the 3 FTx paralogs, we also calculated the pairwise jaccard similarity [110] to measure the similarities and differences of TFs across them.

### Allelic variation in toxin genes

To investigate allelic variation in toxin genes and to design a robust protein database for the proteomics experiments, we mapped the hifi genomic reads and venom-gland RNA-seq reads against the genome to call for variants. The hifi genomic reads were mapped using minimap2 version 2.26 [111], with the parameters pre-designed for hifi reads, and the venom-gland RNA-seq reads were mapped using STAR. Then, low-quality and multi-mapped alignments were removed using samtools version 1.18 [112] by removing alignments with MAPQ lower than 30. The variants were called using BCFtools version 1.18 [112] and filtered to remove variants with quality lower than 20 and read depth lower than 4. We only selected biallelic SNPs to retrieve the toxin allelic variation sequences. To remove redundancy, we clustered 100% identical alleles and toxins using CD-HIT version 4.8.1 [113] with parameters “-c 1.0 -aL 1.0 -aS 1.0”.

### Mass spectrometry imaging

Venom glands were dissected from a captive-bred specimen of *A. lubricus* maintained in the Liverpool School of Tropical Medicine Herpetarium, which is a UK Home Office regulated facility. Venom samples were collected three days prior to euthanasia, which was performed via an overdose of pentobarbital solution. Venom glands were then dissected and processed as described previously [22, 114]. Briefly, glands were fixed in RCL2 (Alphelys, France), dehydrated in ethanol, cleared in xylene, and embedded in paraffin. Seven-micrometer-thick sections were optically imaged before applying matrix ($\alpha$-cyano-4-hydroxycinnamic acid (CHCA), 7 mg/ml in 50% ACN, 0.2% v/v trifluoroacetic acid (TFA)) using a Bruker ImagePrep automated matrix sprayer. The matrix-coated sections were then analyzed using an UltraFlex III TOF-TOF (Bruker) operated in linear positive mode and controlled by FlexControl 3.3.85 (Bruker). We used a small laser to yield a spatial resolution of ~50 μm and suppressed ions up to 980 m/z to minimize matrix signal. FlexImaging 4.0 (Bruker) was used to perform MALDI MSI experiments, acquiring 200 laser shots per raster point. MSI data was visualized and analyzed using FlexImaging 4.1 and SCiLS lab 2024B (SCiLS).

We also measured the spatial correlation, the correlation of co-expression in scRNA data, and the physical distance in the genome of toxins. The spatial correlation (co-occurrence) of 3 FTx was estimated by calculating the pairwise distance between the peaks corresponding to the identified 3 FTx in SCILs Lab. The physical distance across 3 FTx were measured based on the middle position of each gene against the others in the same contig, whereas 3 FTx in distinct contigs were not measured. The co-expression of 3 FTx within cells in the scRNA data was calculated using the expression data from Seurat and the Spearman correlation method. The correlations between those measures (i.e., spatial correlation, physical distance, and co-expression correlation) were estimated using the Pearson correlation test in R.

### Top-down venom proteomics

Denaturing top-down proteomic experiments were performed as previously described [68]. Briefly, the collected venom sample was dissolved in liquid chromatography-mass spectrometry (LC-MS) grade water to a final concentration of 10 mg/mL, and centrifuged at 12,000 × g for 5 min. For reduction of disulfide bonds, 10 μL of dissolved venom was mixed with 10 μL of 0.5 M TCEP (tris(2-carboxyethyl)phosphine), and 30 μL of 0.1 M citrate buffer (pH 3). After 30 min incubation at 65 °C, samples were mixed with 50 μL of acetonitrile/formic acid/H_2_O (10:1:89, v/v/v) and centrifuged at 12,000 × g for 5 min. After centrifugation, 5 μL of the supernatant of reduced samples was injected for LC-MS/MS analyses. LC-MS/MS experiments of two technical replicates were carried out on a Vanquish ultra-high-performance liquid chromatography (UHPLC) system coupled to a Q-Exactive HF quadrupole orbitrap (Thermo Fisher Scientific, Bremen, Germany). LC separation was performed on Supleco Discover BIO Wide Pore C18, 150 $\times$ 2.1 mm, 3 μm particle size, 300 A pore size column, (Sigma-Aldrich, USA) at a temperature of 30 °C. A flow rate of 0.5 mL/min was used and the samples were eluted with a gradient of LC-MS grade water with 0.1% formic acid (FA) and 0.1% FA in LC-MS grade acetonitrile (ACN). Peptides were eluted across a 25-min linear gradient from 5 to 40% ACN followed by 40–70% ACN across 5 min.

ESI settings of the mass spectrometer were adjusted to 50 AU sheath gas, 13 AU auxiliary gas, spray voltage 3.5 kV, S lens RF level 70 V, and capillary temperature 363 °C. The MS1 acquisition included a resolution of 240 K, AGC target set at 1e6, maximum Injection Time was set at 1000 ms and a scan range set from 300 to 3500 m/z was acquired. MS2 spectra were obtained in data dependent acquisition (DDA) mode. Mass spectra were acquired with 1 microscan and 200 ms maximal C-trap fill time. AGC targets were set to 5E5 for MS/MS scans. A resolution of 240 K (at m/z 200) was used also for MS2 acquisition. The three most abundant ions of the survey scan with known charge were selected for fragmentation into the higher-energy C-trap dissociation (HCD) with a normalized collision energy (NCE) stepped through 25%, 30%, and 35%. An isolation window of 3.0 m/z was used for MS1 precursor ion selection. The apex trigger was set activated with the MS2 event occurrence set within 2 to 30 s from the Full Scan (MS1) peak. Dynamic exclusion was enabled to prevent the same precursor ion from being selected within a 30-second window. For data analysis, the raw LC-MS/MS data was converted to.mzML file format using MSconvert of the ProteoWizard package version 3.0.21339-f15 d0fc. For protein spectrum matching, multiple charged MS/MS spectra were then deconvoluted using Top-FD version 1.7.3 [115]. For the deconvolution, the maximum charge was set to 30, maximum mass was set to 70,000, signal-to noise threshold was set to 3 for MS1 and 1 for MS2. The protein spectrum matching was performed using TopPIC version 1.7.3 [116] against the peptide sequences from the final protein-coding gene annotation set of *A. lubricus*. Basic parameters were a max variable PTM number set at 3 and the Mass error tolerance (PPM) set at 10. The cut-off settings spectrum level and the proreform level set were kept to an e-value cut-off of 0.01.

## Supplementary Information

Additional file 1.

Additional file 2.

Additional file 3.

## Data Availability

The genome assembly and the data generated in the present study are available in the NCBI under the project number PRJNA821017 [117]. Genome assembly and final gene annotation set are also available in the figshare database [118]. Raw mass spectrometry data is available in the MassIVE under dataset number MSV000096349 [119]. A list with all additional datasets used in the present study is available in Additional file 2: Table S10. No custom code was used.
