# Supplementary material for: The gene regulatory mechanisms shaping the heterogeneity of venom production in the Cape coral snake
Source: Genome Biol. 2025 May 19;26:130. doi: 10.1186/s13059-025-03602-w (PMC12087220; doi:10.1186/s13059-025-03602-w)
Supplement: Supplementary file 1 — Additional file 1. [file 13059_2025_3602_MOESM1_ESM.pdf]

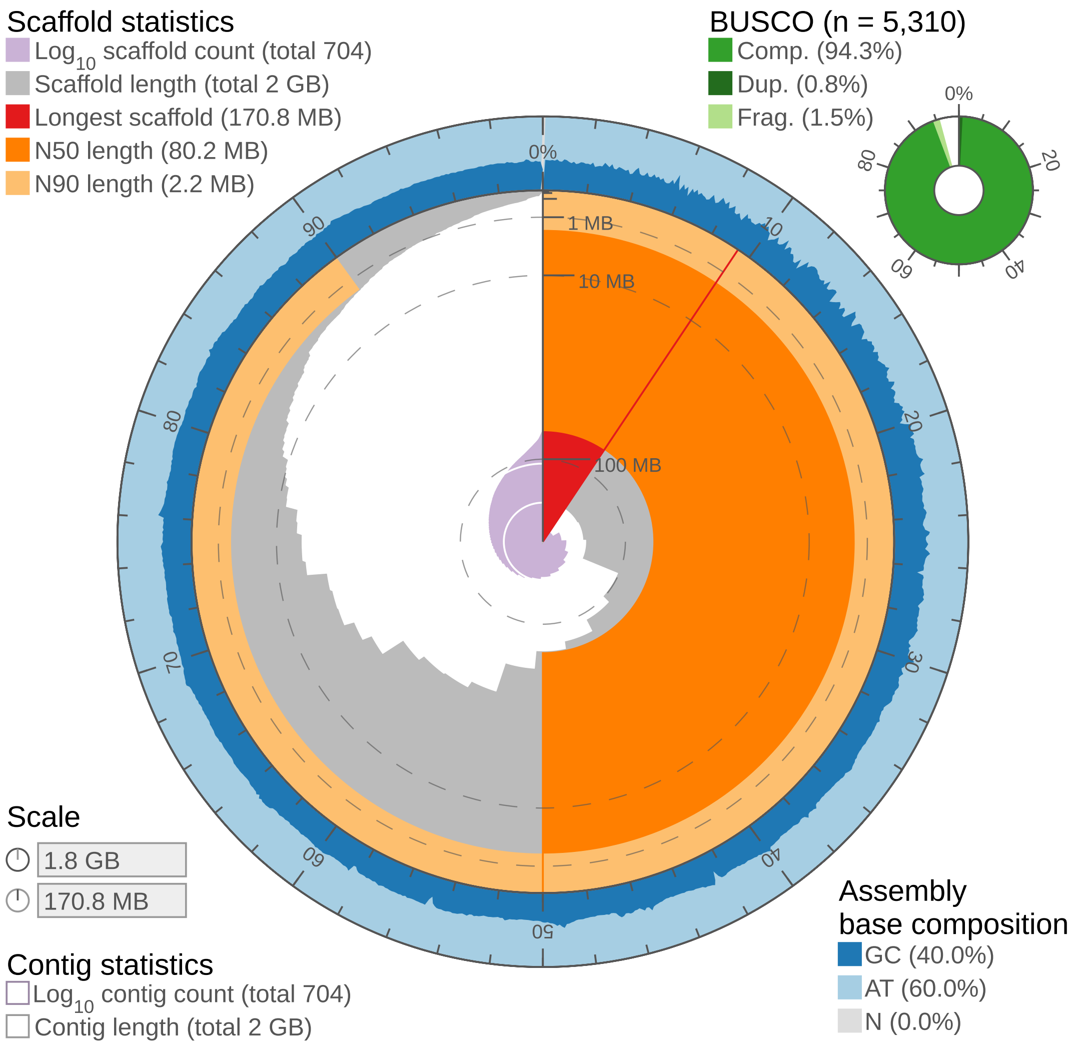

Figure S1: Snail plot summarizing metrics of the genome assembly obtained for *Aspidelaps lubricus*. The longest contig is represented with the red line (∼170.8 Mb). The N50 is represented in dark orange color (80.2 Mb). The N90 is represented in light orange color (2.2 Mb). The base composition is represented in light and dark blues at the outer circle (∼40% of GC content). The BUSCO completeness is represented in the top right circle, which revealed 94.3% of complete genes (93.5% single-copy and 0.8% duplicated), 1.5% of fragmented genes, and 4.2% of missing genes, when using the vertebrate database odb10, which contains 5,310 genes. All metrics revealed a high-quality genome assembly.

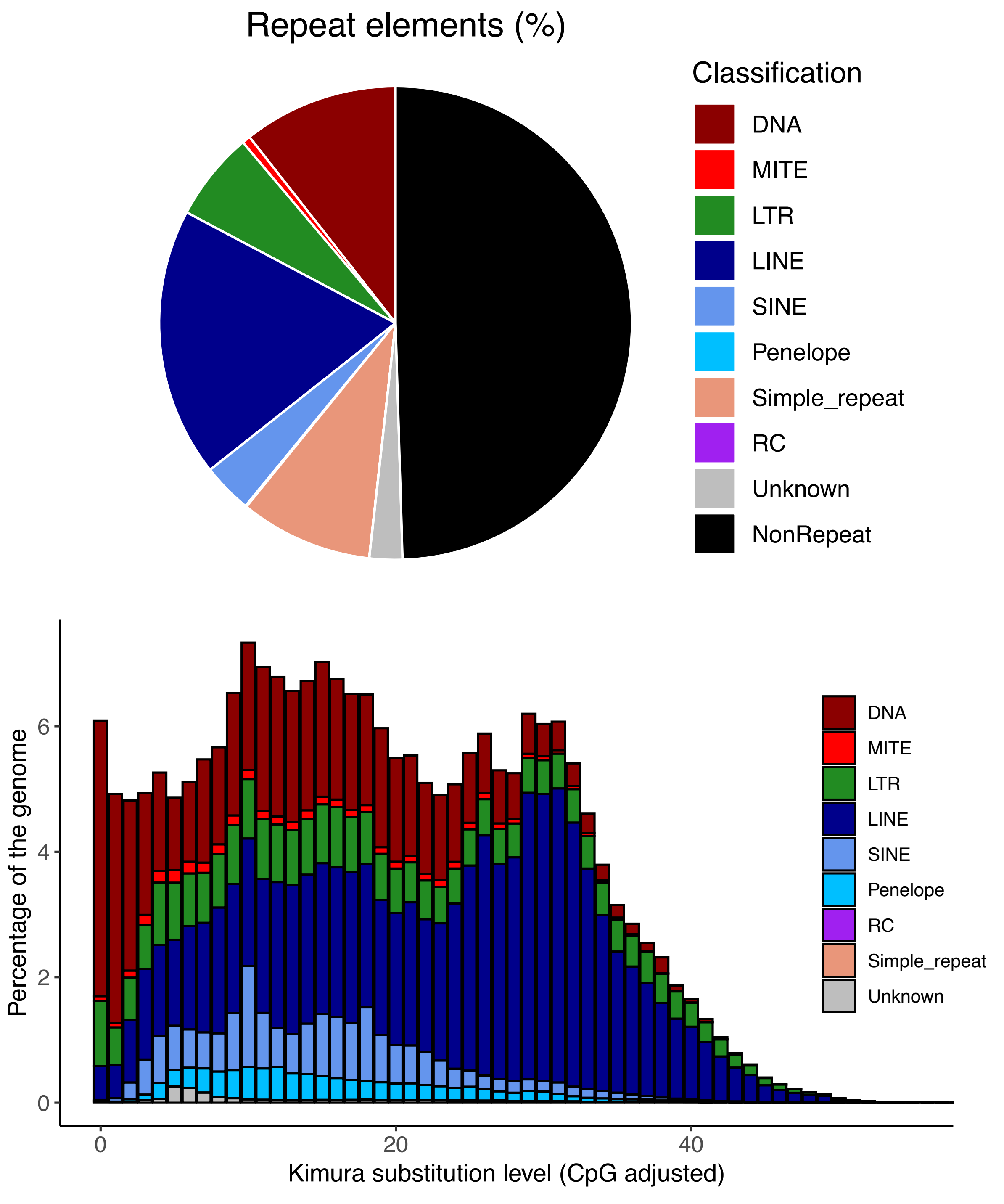

Figure S2: Repeat landscape of *Aspidelaps lubricus* genome. The piechart at the top shows the percentage of repeat elements annotated in the genome assembly, which reveals a high percentage of LINE elements as expected for squamates. The barplot at the bottom shows the repetitive landscape obtained for the genome assembly through calculation of the Kimura substitution levels. The x-axis represents the percentage of the genome matching to repeat sequences. The y-axis represents the divergence of repeat sequences to the consensus, whereas the left bars indicate recent expansions and and right bars indicate ancient expansions. This analysis suggests recent bursts of DNA transposons and ancient bursts of LINE elements.

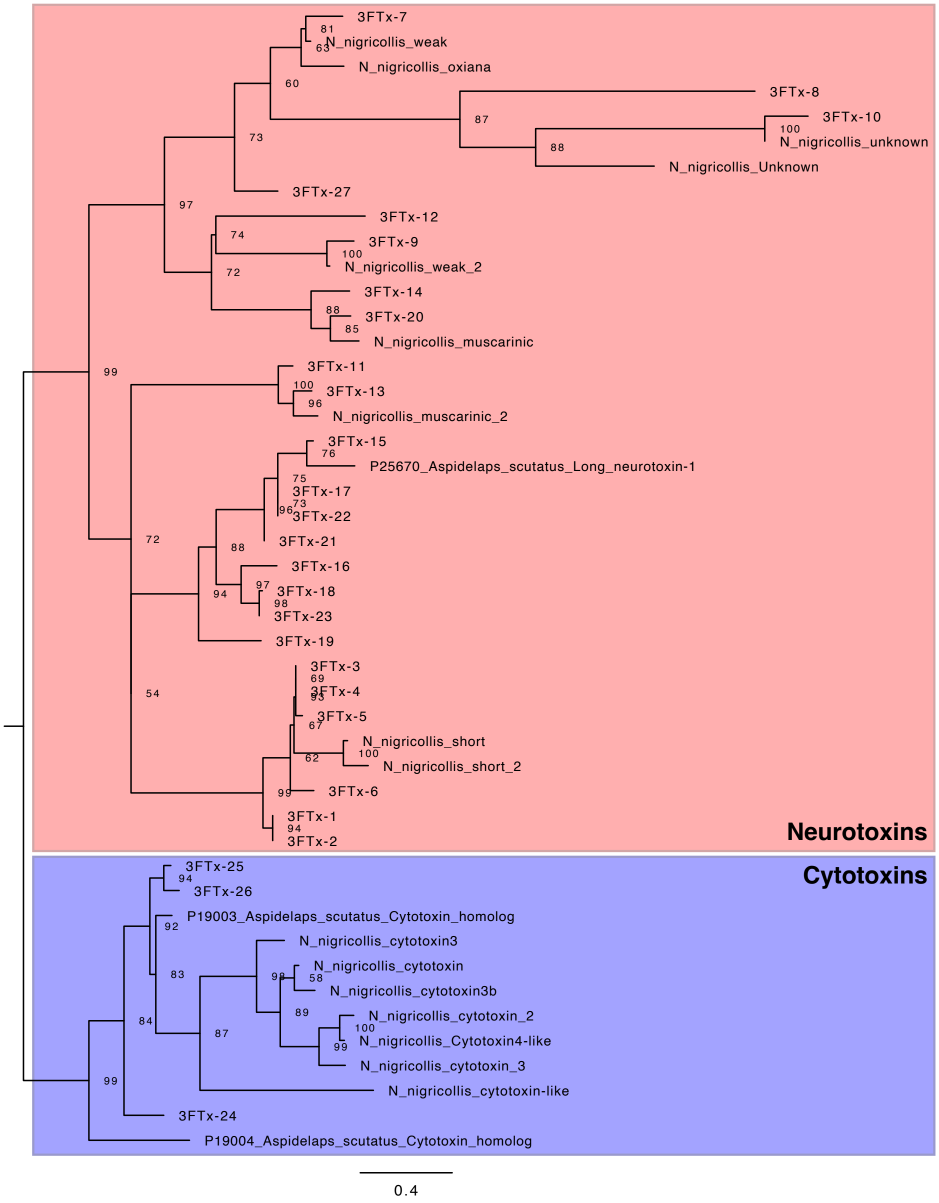

Figure S3: Phylogenetic tree of 3FTx genes annotated in *Aspidelaps lubricus* genome including known 3FTx peptides from the closely related species *A. scutatus* and *Naja nigricollis*. The support values of bootstrap are shown at tree nodes. This analysis revealed that the 3FTx-24, -25, and -26 are cytotoxins (highlighted in blue), whereas all other 3FTx are neurotoxins (highlighted in red).

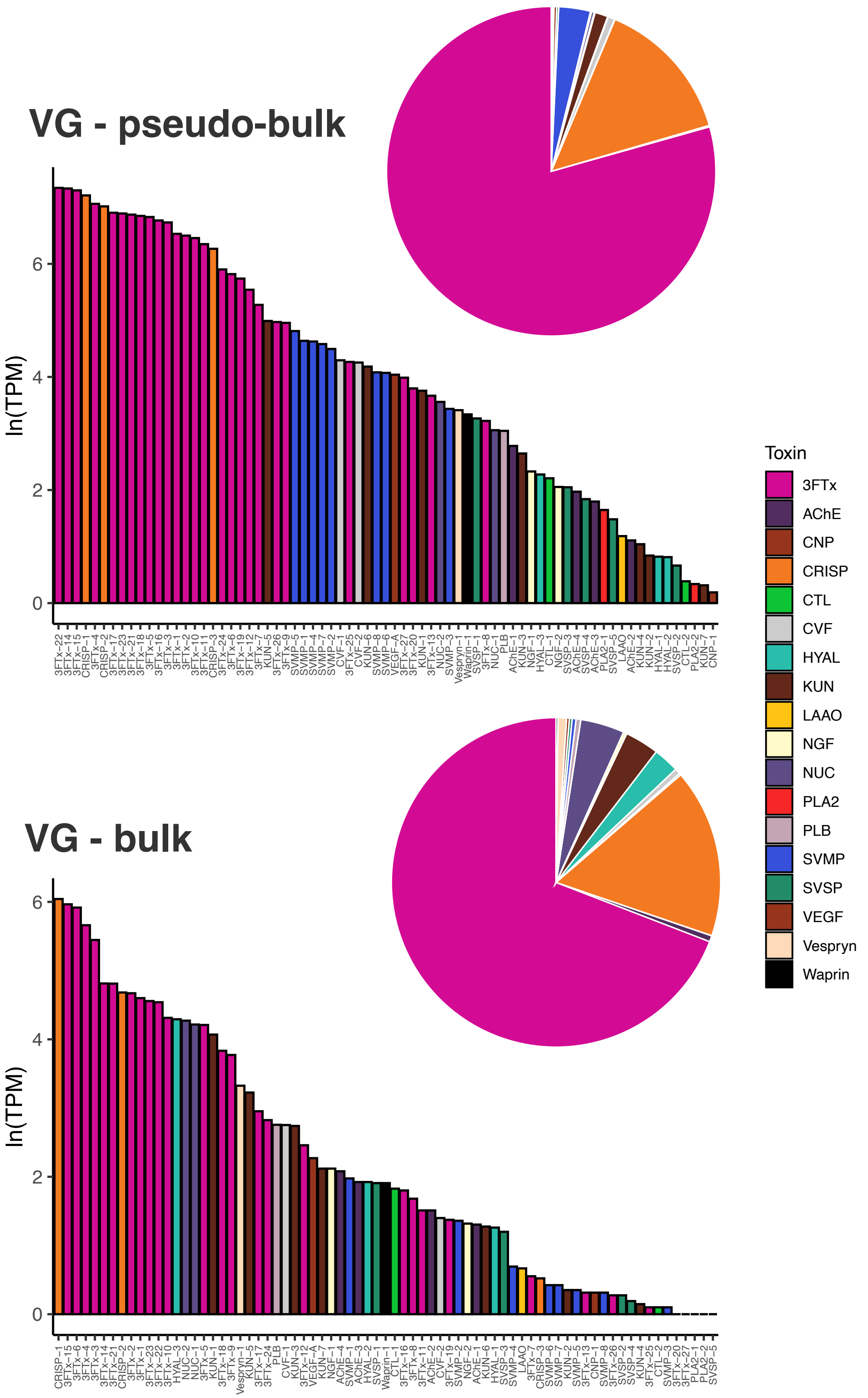

Figure S4: Toxin expression profile of venom gland tissue using the scRNA-seq data (pseudo-bulk) and the whole tissue RNA-seq data (bulk). It reveals that the major components of toxin expression of *Aspidelaps lubricus* are 3FTx.

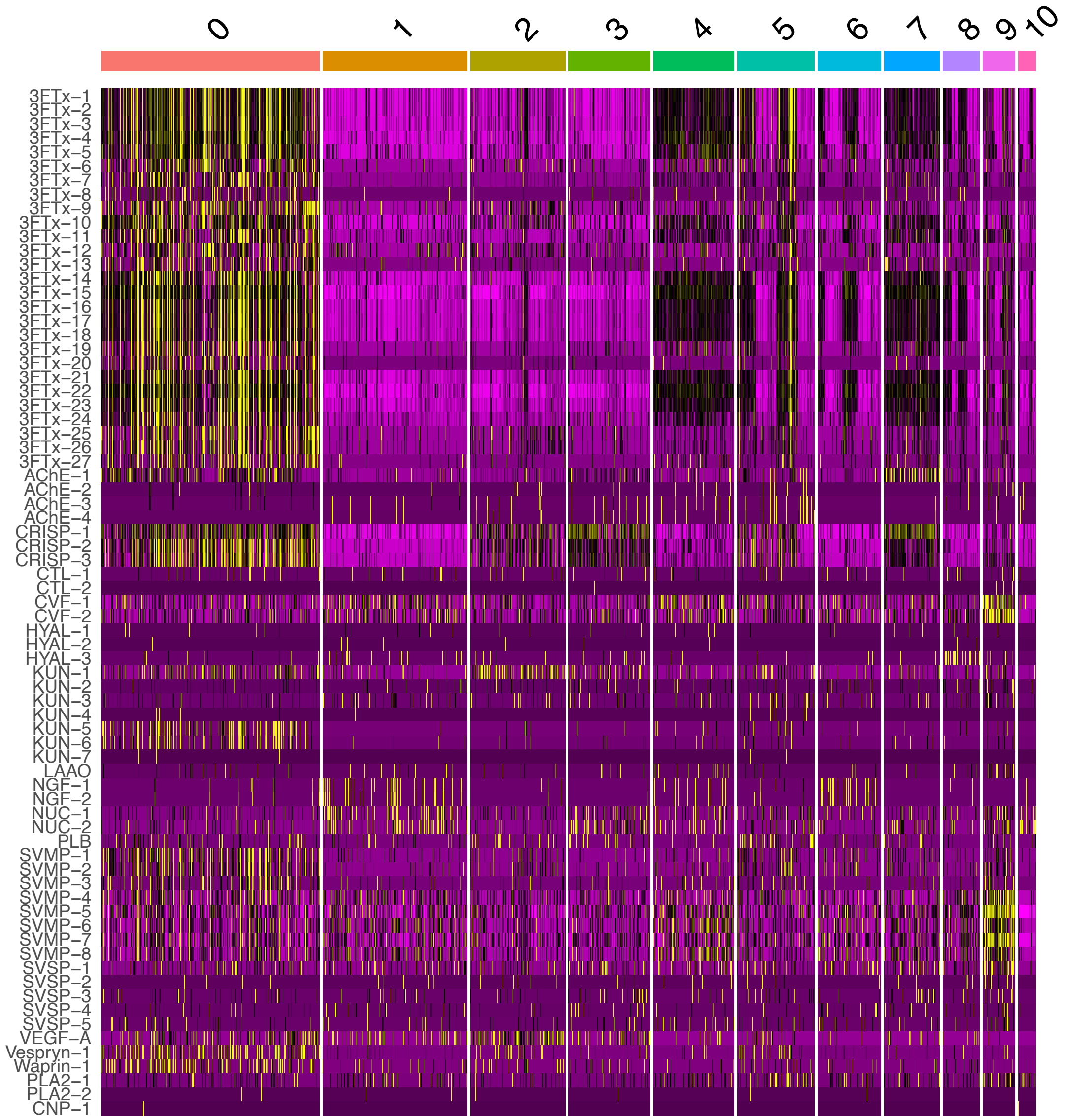

Figure S5: Heatmap of toxin expression (x-axis) per individual cell (x-axis). Yellow represents high expression level, black represents the middle point between high and low expression level, and light purple represents low expression level. The cells from toxin-enriched clusters 0, 4, 5, 7 and 9 display expression of most abundant toxins (see Figure S4), which indicates those clusters as the toxin producing cells.

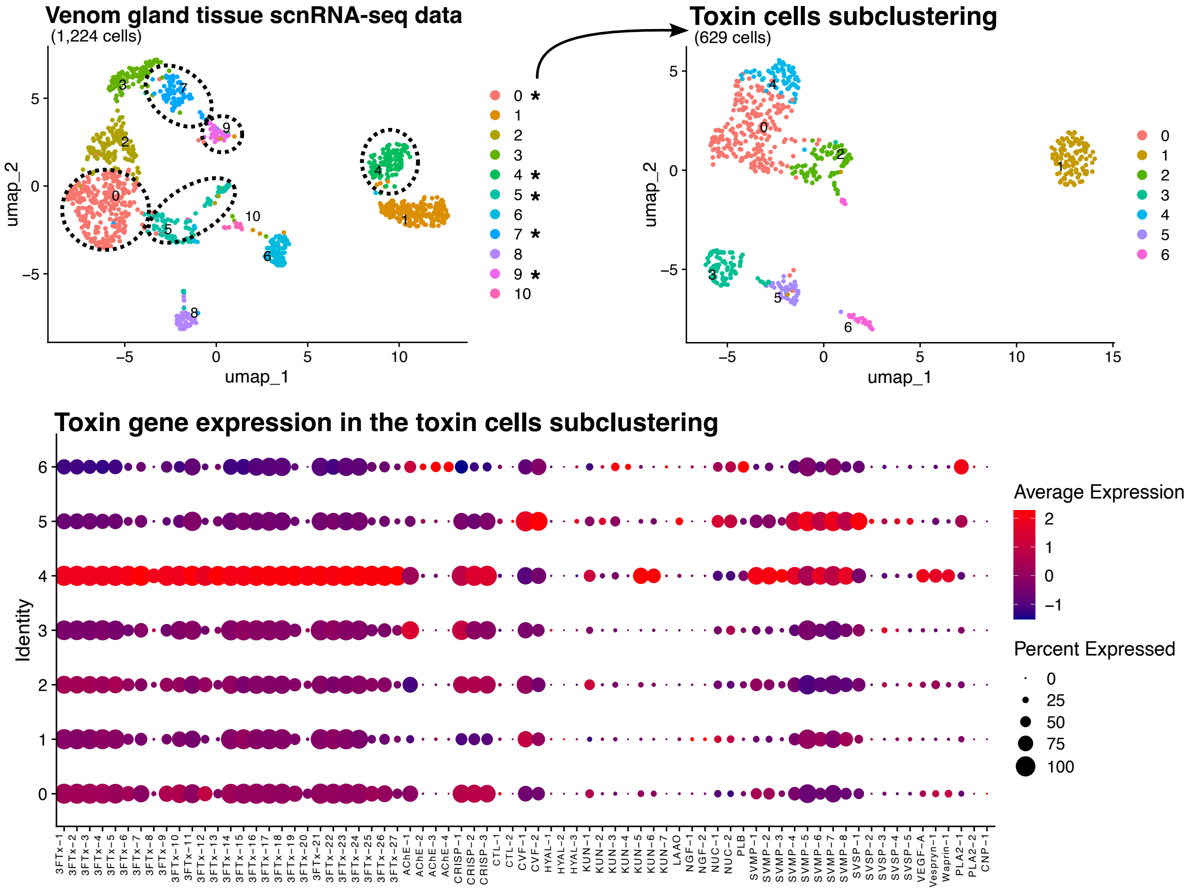

Figure S6: Subclustering of toxin cells (clusters 0, 4, 5, 7, and 9; total of 629 cells; marked with asterisks "*" and dashed lined circles) from the total venom gland tissue dataset (11 clusters; total of 1,224 cells). The visualization is shown using UMAP for both clustering and subclustering. At the bottom, the expression profile of toxin genes in each cell cluster obtained after subclustering. The colors represent the average expression of that toxin in that cluster (with red representing higher expression and dark purple representing lower expression). This analysis revealed subclusters with heterogeneous toxin expression profiles.

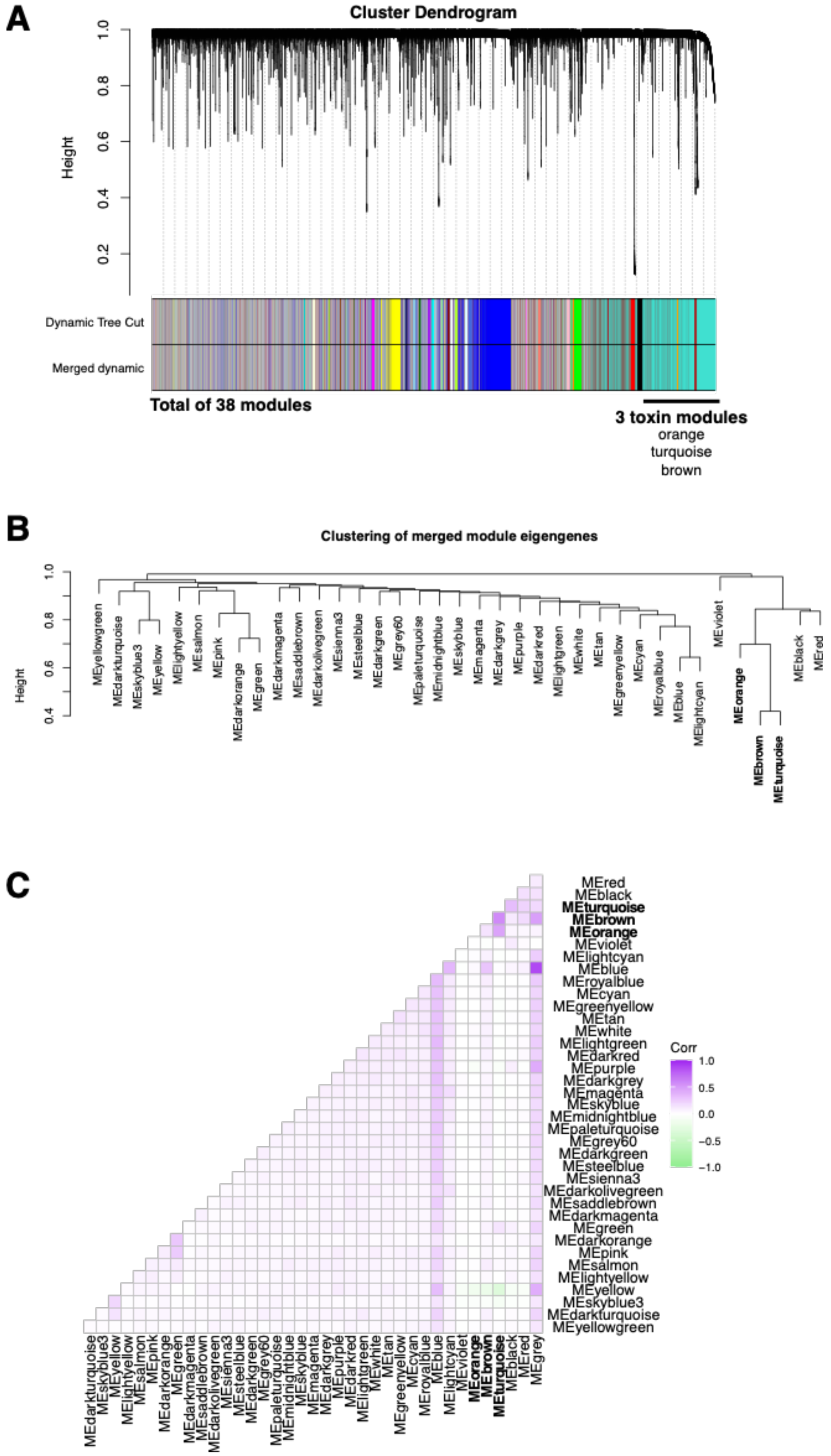

Figure S7: Weighted gene co-expression network analysis (WGCNA) using the 1,224 cells of the venom gland of *A. lubricus*. (A). Clustering of genes showing the 38 modules identified through WGCNA highlighting the 3 toxin modules. (B) Clustering of modules showing the correlation between the 3 toxin modules. (C) Correlation plot of modules identified in the WGCNA, of which higher correlation is represented with dark purple colors. The toxin modules are highlighted in bold.

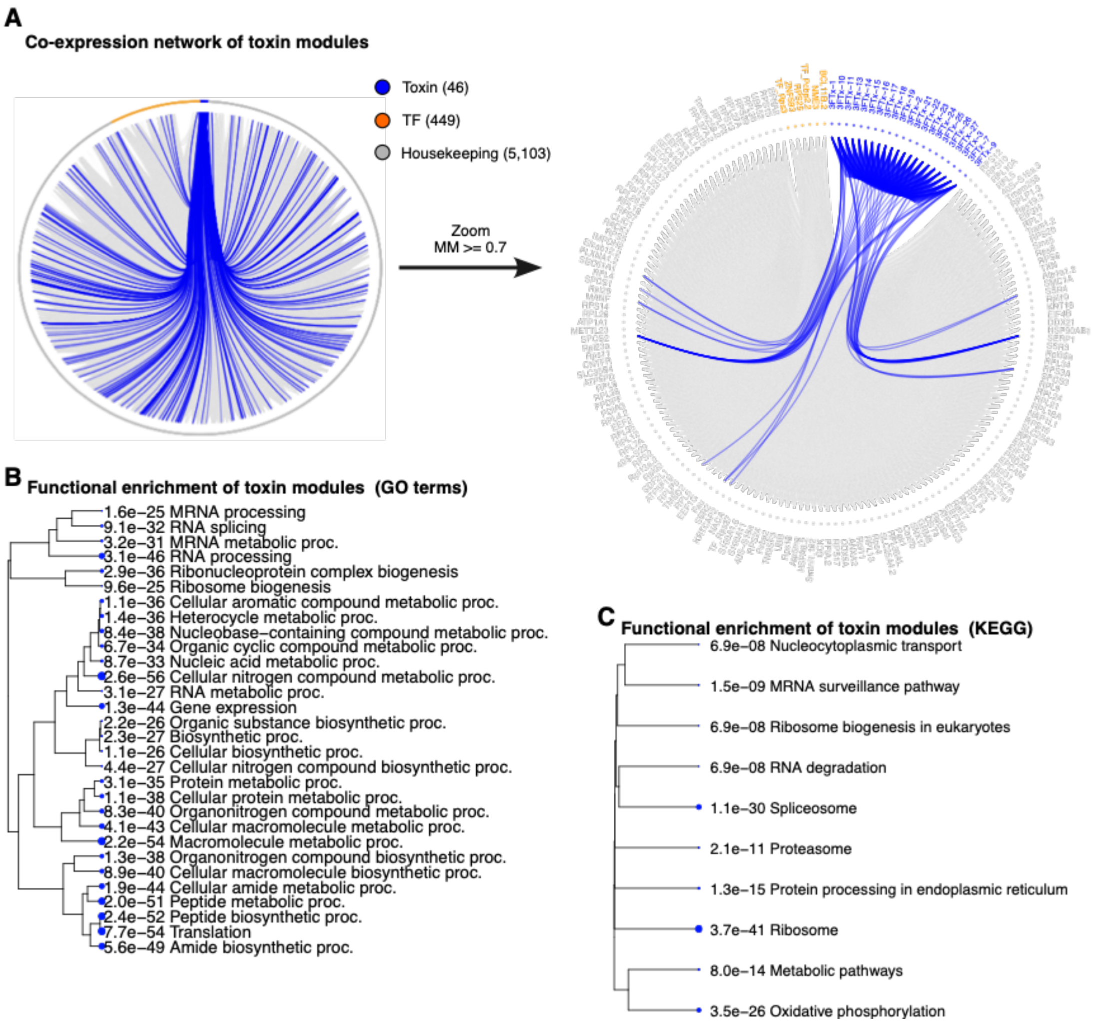

Figure S8: Modules of co-expression of 1,224 cells from the venom gland of *A. lubricus*. (A) The weighted gene co-expression network of toxin modules using toxin cells comprised 5,598 genes. Of these 46 were toxins (blue), 449 were transcription factors (orange), and 5,103 were housekeeping (gray). On the left, a network with all genes within toxin modules. On the right, a zoom in showing genes filtered to have module membership (MM) greater or equal to 0.7 and adjacency greater than 0.01 for better visualization purposes. In both networks, the edges linking to toxin genes are highlighted in blue. (B) The 30 most significant GO terms of biological processes enriched in the toxin modules. (C) The 10 most significant KEGG pathways enriched in the toxin modules. Those pathways are shown based on their relationships of GO terms and the calculated p-values are shown before the GO names.

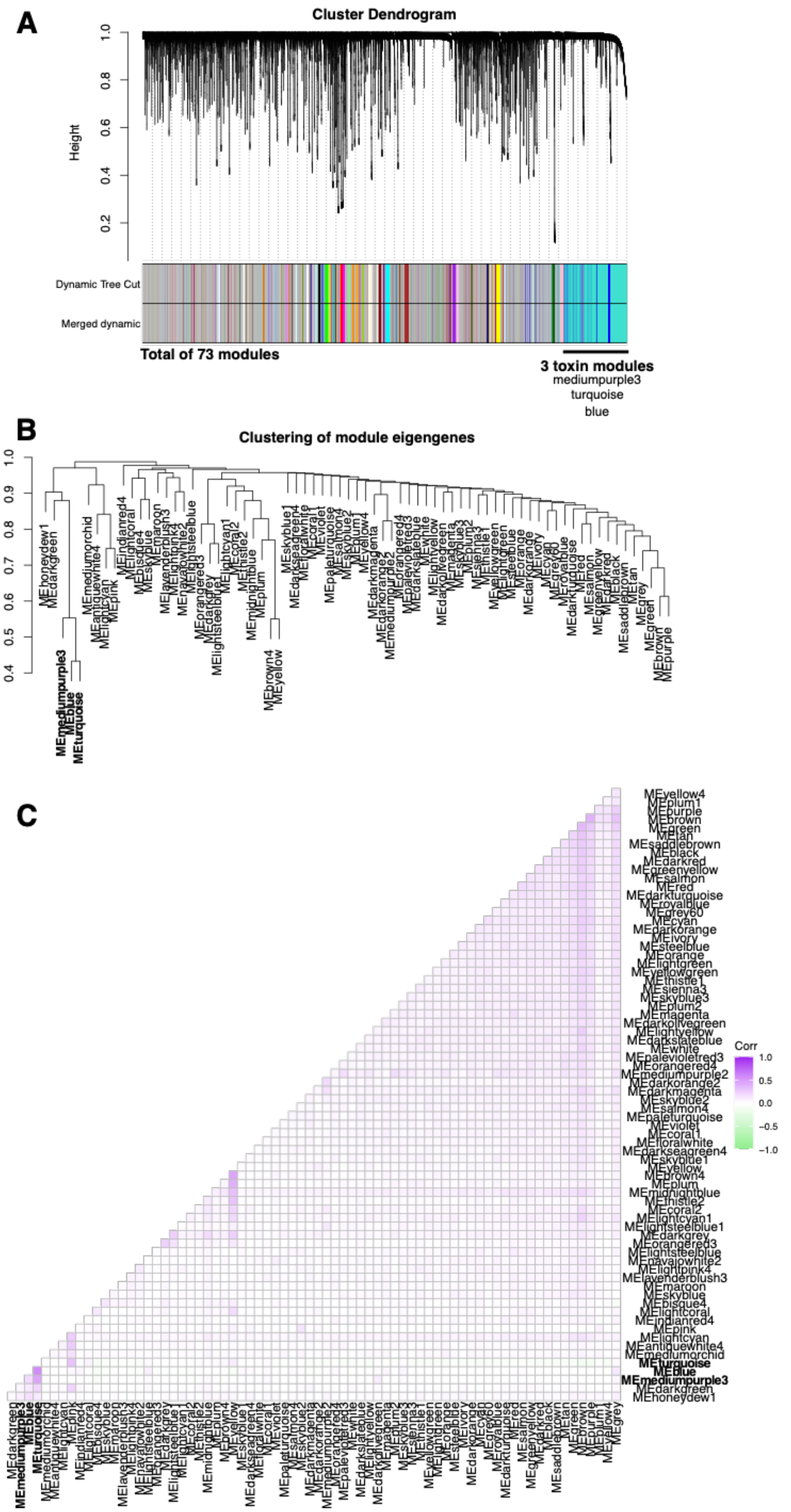

Figure S9: Weighted gene co-expression network analysis (WGCNA) using the 629 toxin cells (see Figure 2 and Figure S6). (A). Clustering of genes showing the 73 modules identified through WGCNA highlighting the 3 toxin modules. (B) Clustering of modules showing the correlation between the 3 toxin modules. (C) Correlation plot of modules identified in the WGCNA, of which higher correlation is represented with dark purple colors. The toxin modules are highlighted in bold.

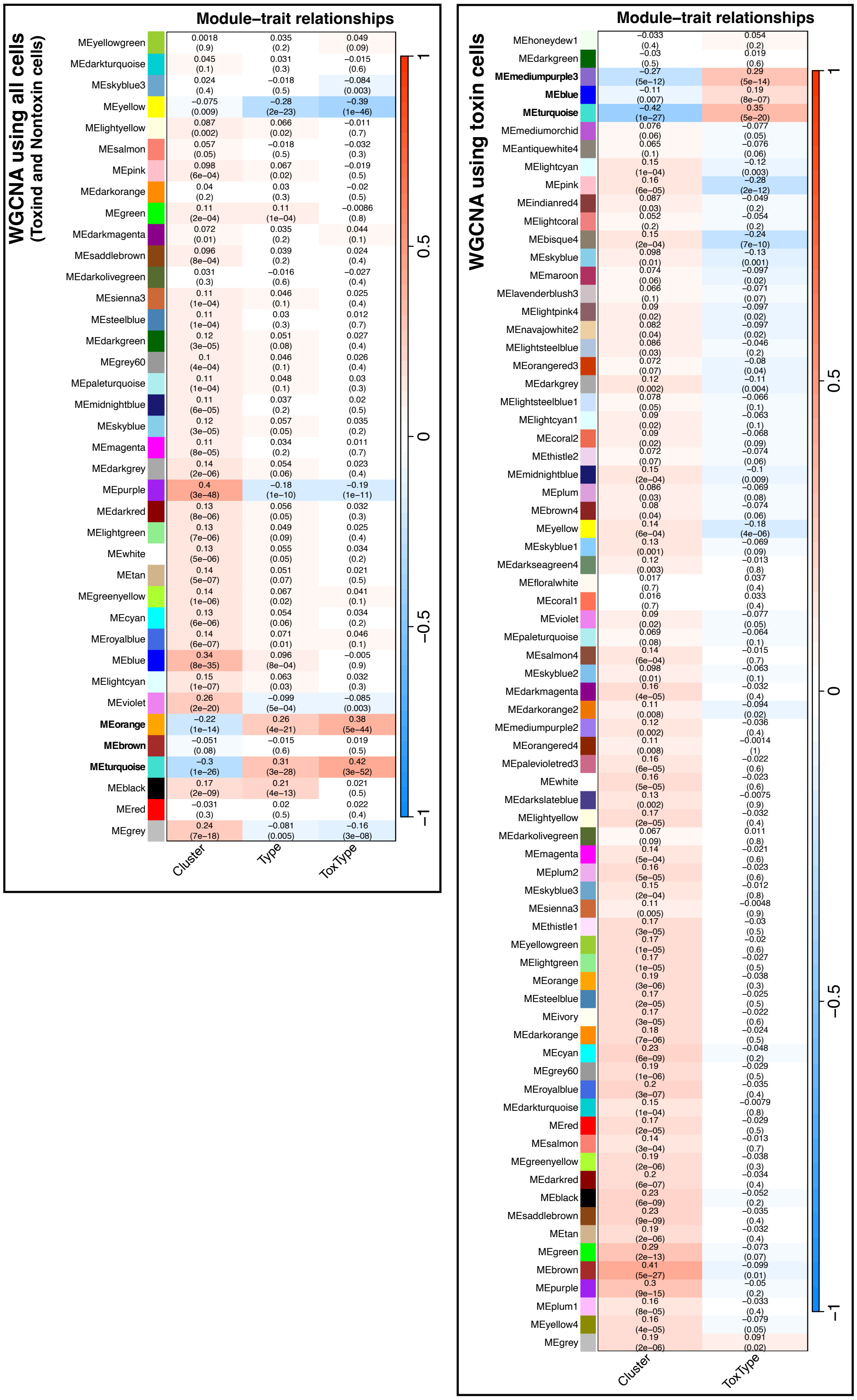

Figure S10: Consensus network modules correlated with cells classification using the eigenmodule (the first principal component of the module) in both WGCNA analysis using all 1,224 cells (at left) and the 629 toxin cells (at right). Correlation coefficient along with p-value in parenthesis underneath and color-coded according to correlation coefficient. "Cluster" means the clusters obtained in the clustering step of scRNA-seq analysis. "Type" in the left chart means toxin or nontoxin cells (see Figure 2). "ToxType" means cells producing 3FTx, SVMP, or other toxins as obtained in the subclustering step of scRNA-seq analysis (see Figure S6). The toxin modules are highlighted in bold.

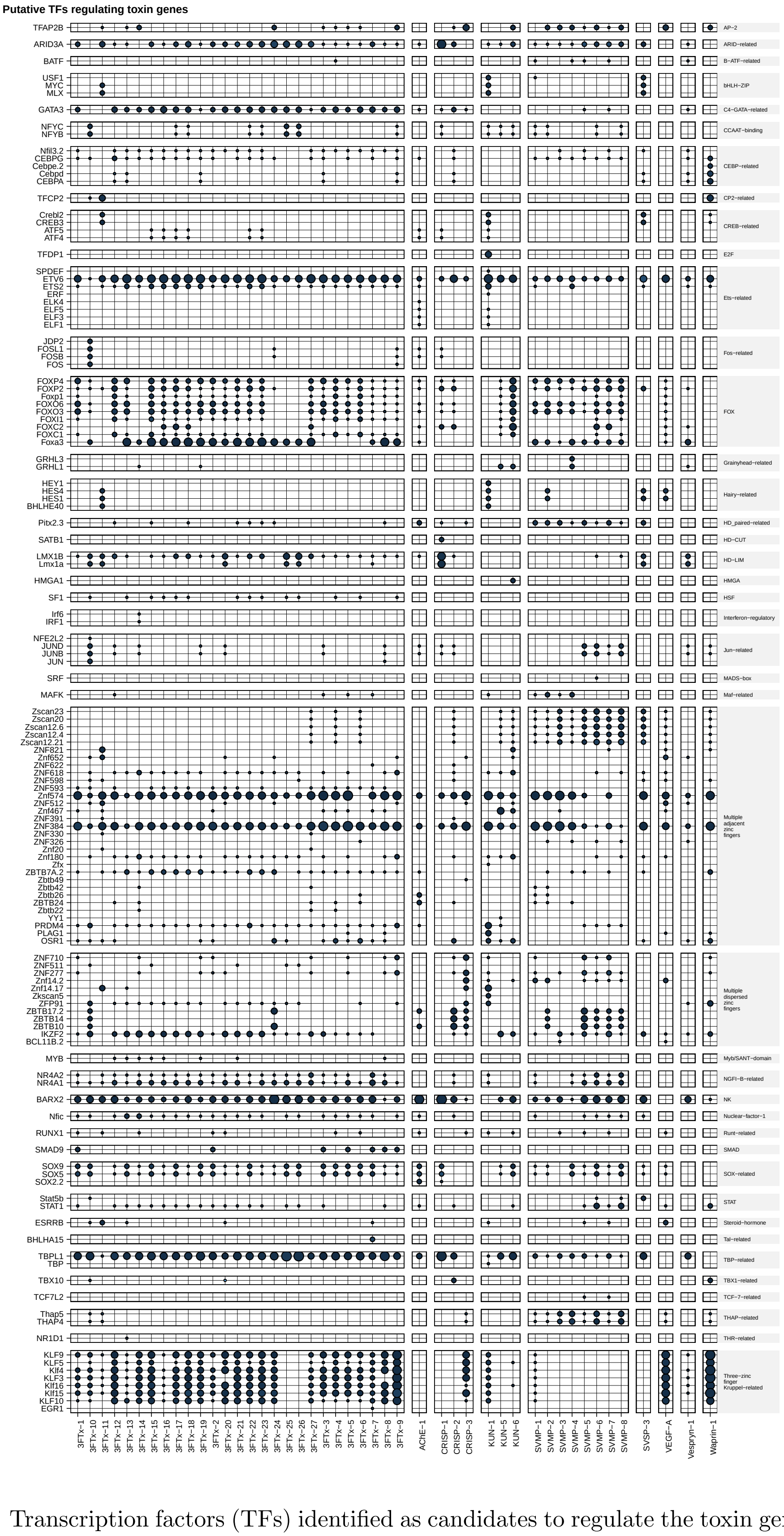

Figure S11: Transcription factors (TFs) identified as candidates to regulate the toxin gene expression using all 1,224 venom-gland cells. Rows correspond to the TFs homologous to profiles at JASPAR in toxin modules and columns correspond to toxin genes in the toxin modules. Circles indicate transcription factor binding sites (TFBSs) in the core promoter of the toxin gene. The size corresponds to the number of predicted TFBSs in a given promoter, in which larger circles represent more bound sites. The color corresponds to the network adjacency weights calculated using GENIE3, in which lighter colors represent higher weights.

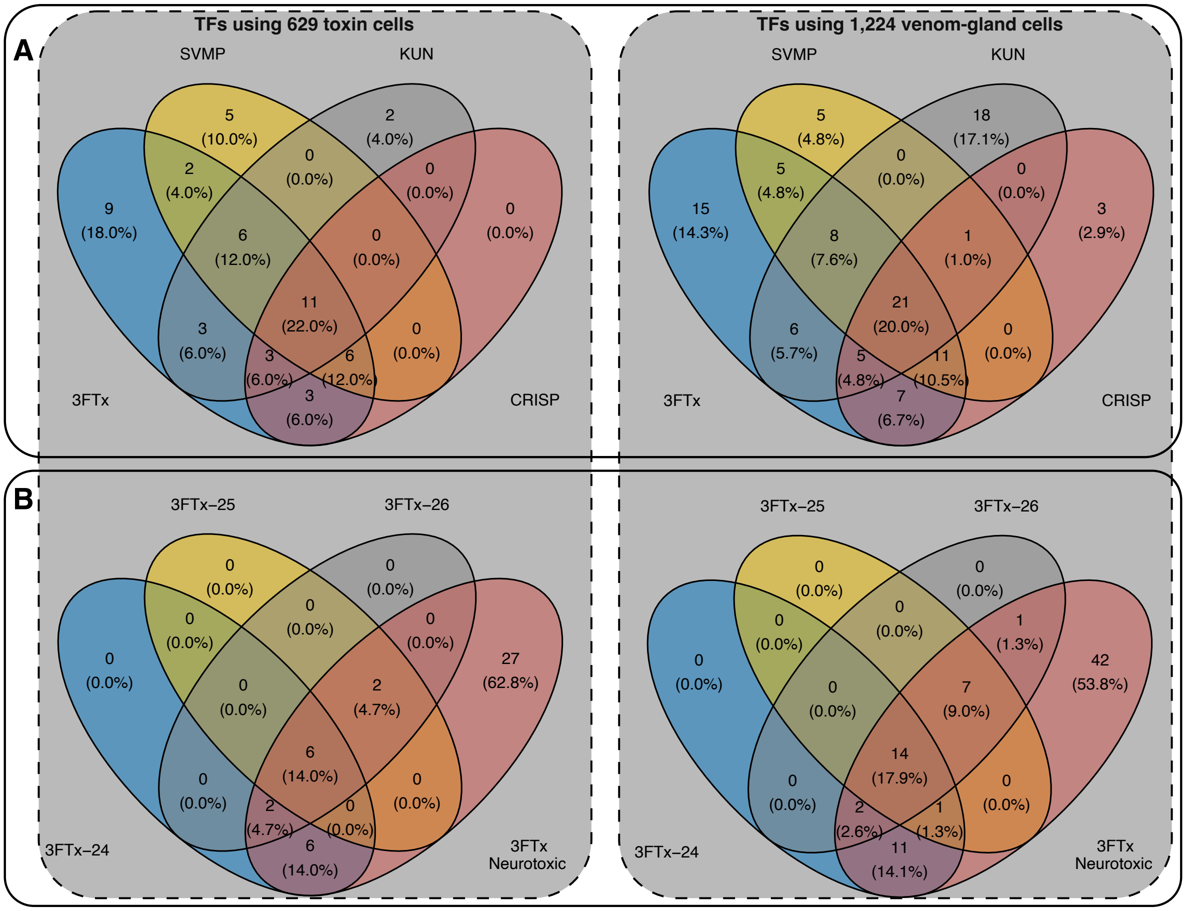

Figure S12: Venn diagram of candidate TFs regulating toxin genes. (A) Overlap of candidate TFs regulating the toxin families with major abundance in venom-gland transcriptome (i.e., 3FTx, SVMP, KUN, and CRISP). (B) Overlap of candidate TFs regulating the cytotoxic (3FTx-24, 3FTx-25, and 3FTx-26) and neurotoxic paralogs of 3FTx. The analysis obtained using 629 toxin cells is shown at the left, whereas the analysis using 1,224 venom-gland cells is shown at the right.

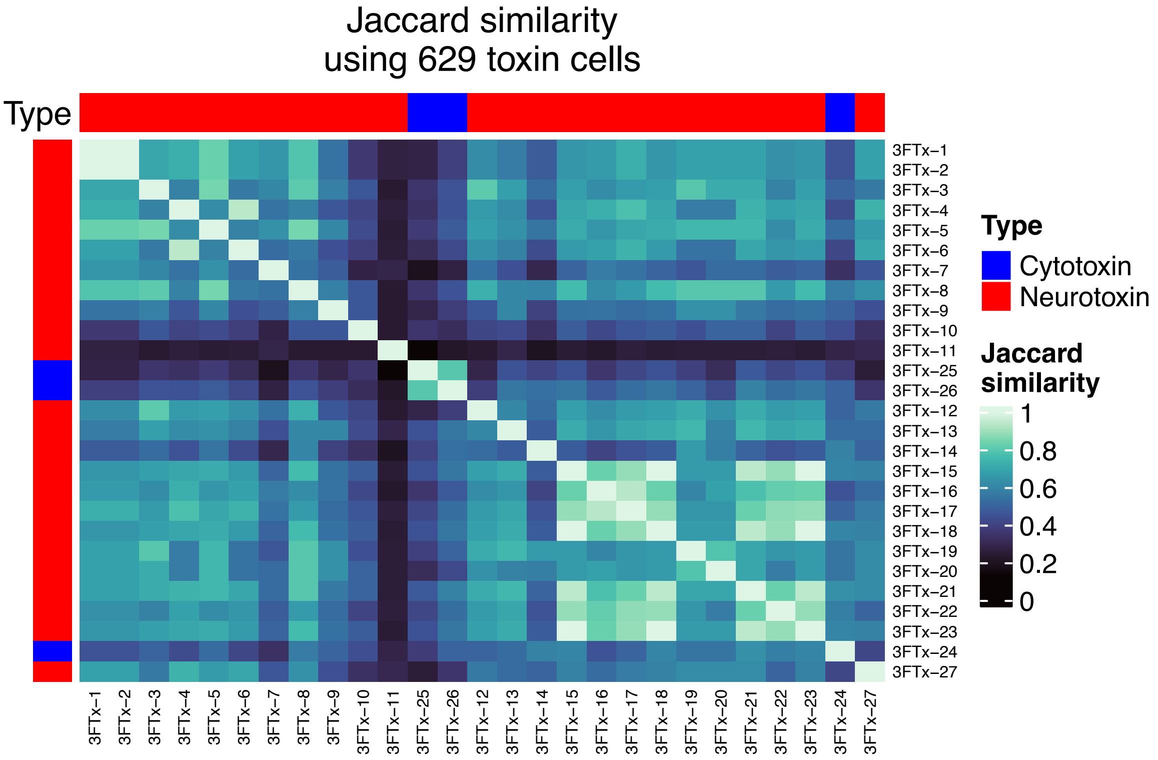

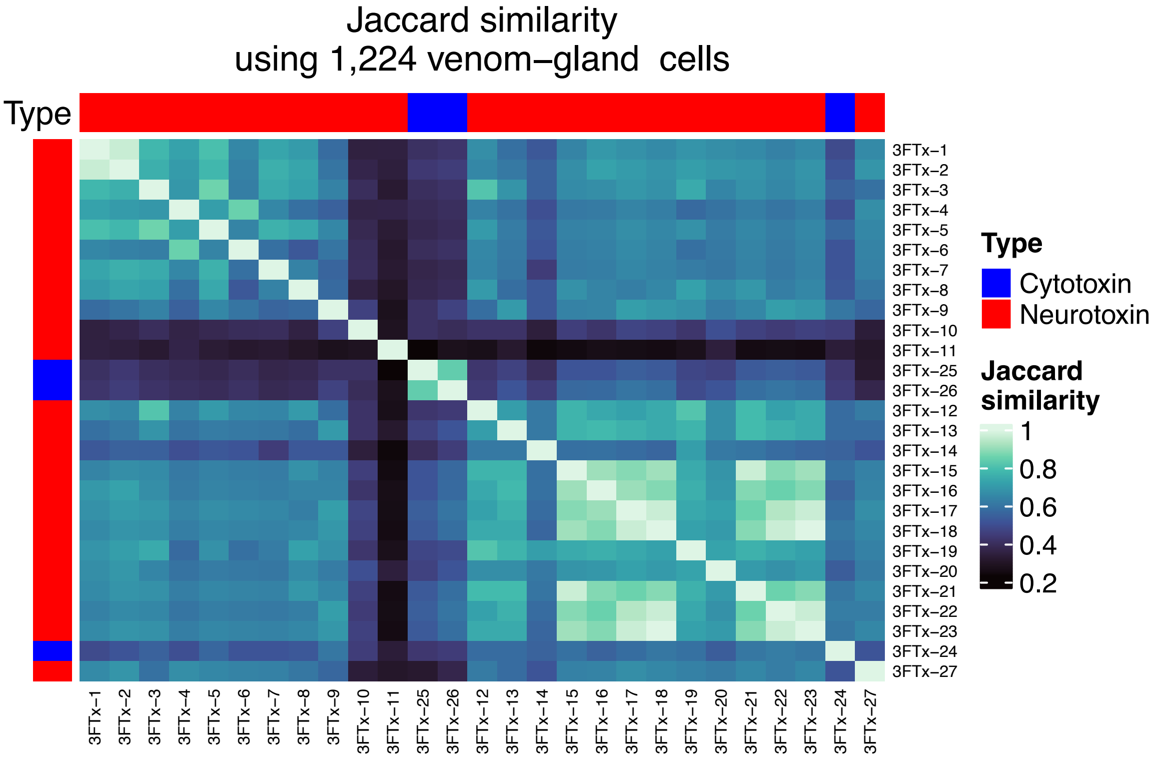

Figure S13: Pairwise jaccard similarity across 3FTx genes. Lighter colors indicate high similarity, whereas darker colors indicate low similarity. This measure allowed us to understand the similarity of 3FTx paralogs when analyzed through their candidate TFs. The analysis obtained using 629 toxin cells is shown at the top, whereas the analysis using 1,224 venom-gland cells is shown at the bottom.

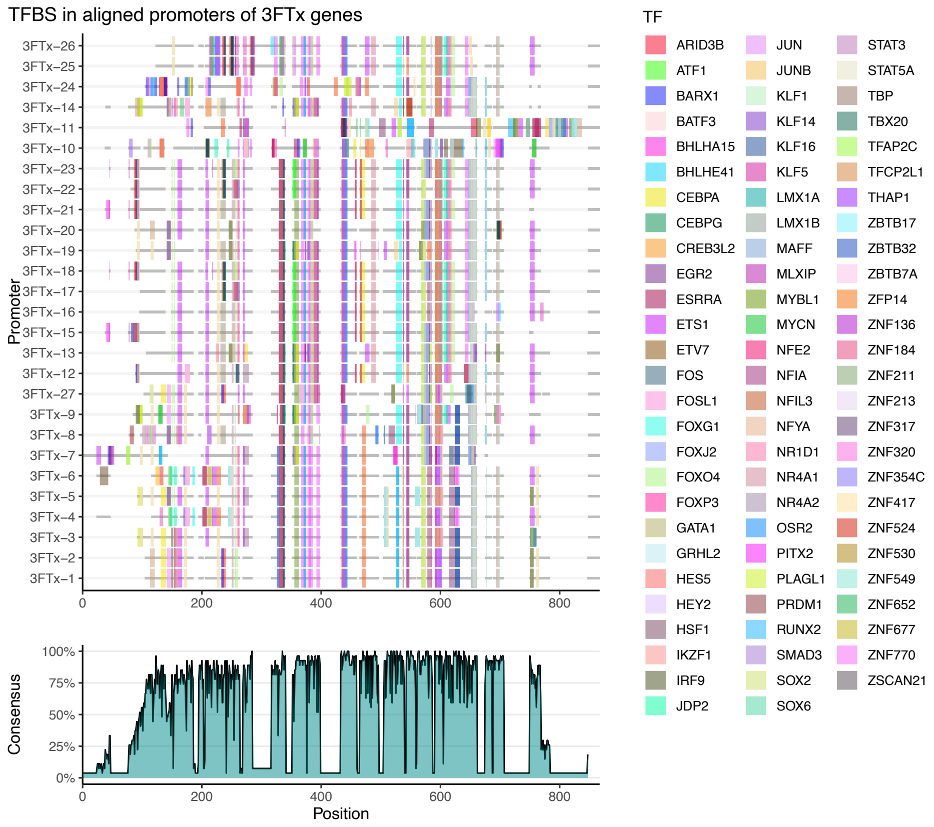

Figure S14: Alignment and conservation of promoter sequences of 3FTx with the TFBSs identified based on the 1,224 venom-gland cells. The gray regions represent alignment gaps. The cytotoxins are highlighted in bold (i.e., 3FTx-24, 3FTx-25, and 3FTx-26).

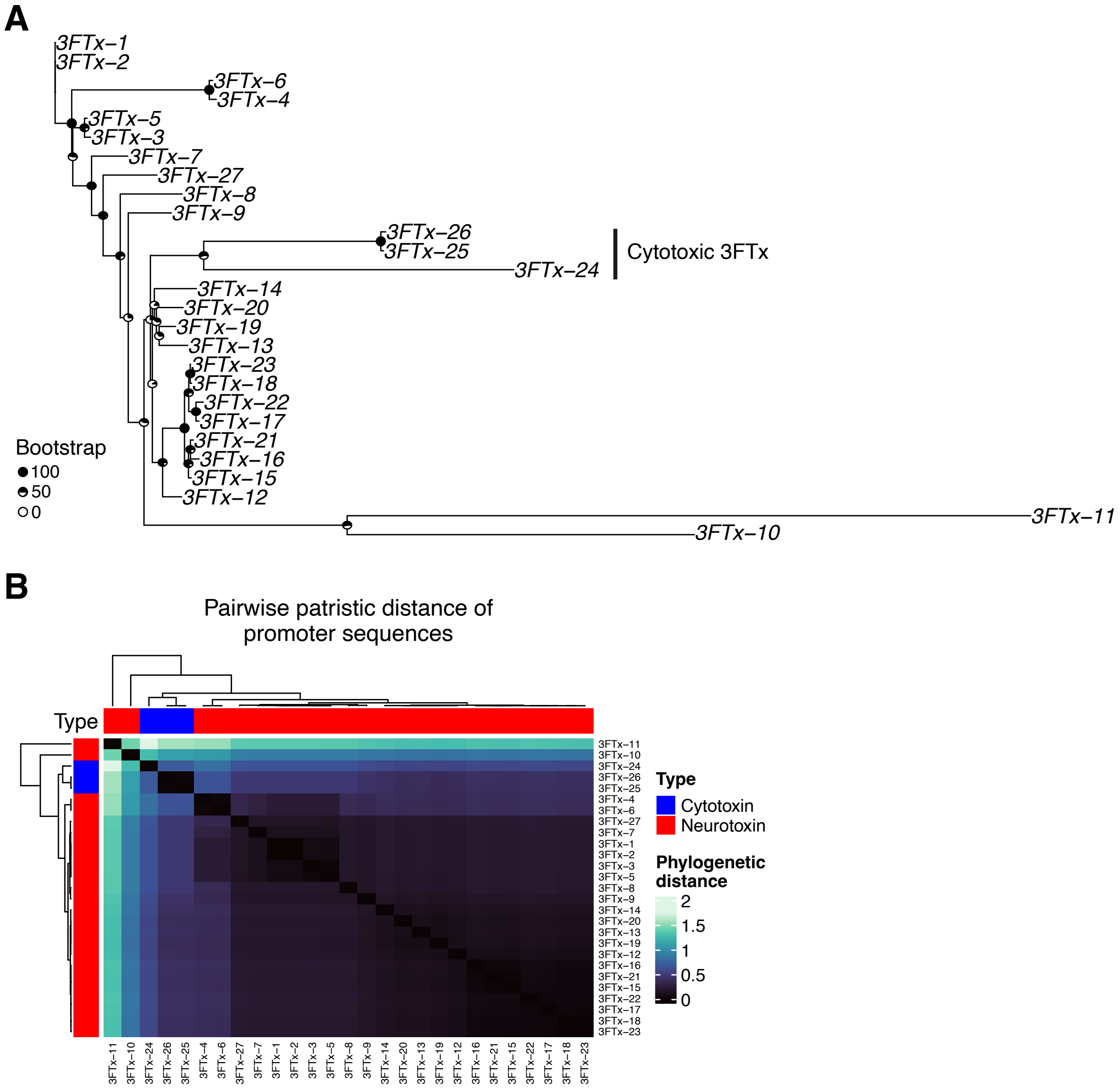

Figure S15: Phylogenetic relationships of core promoter sequences of 3FTx genes. (A) Phylogenetic tree of core promoter sequences. The support values of bootstrap are shown at tree nodes. This analysis revealed that the core promoters of cytotoxic types of 3FTx are closely related when compared to the neurotoxic types. As previously observed using the mature peptide in Figure S3. (B) Pairwise phylogenetic (patristic) distance of 3FTx genes when analyzing their core promoter sequences. Lighter colors indicate higher phylogenetic distance, whereas darker values indicate lower phylogenetic distance. The columns and row annotations indicate the neurotoxins (in red) or cytotoxins (in blue).

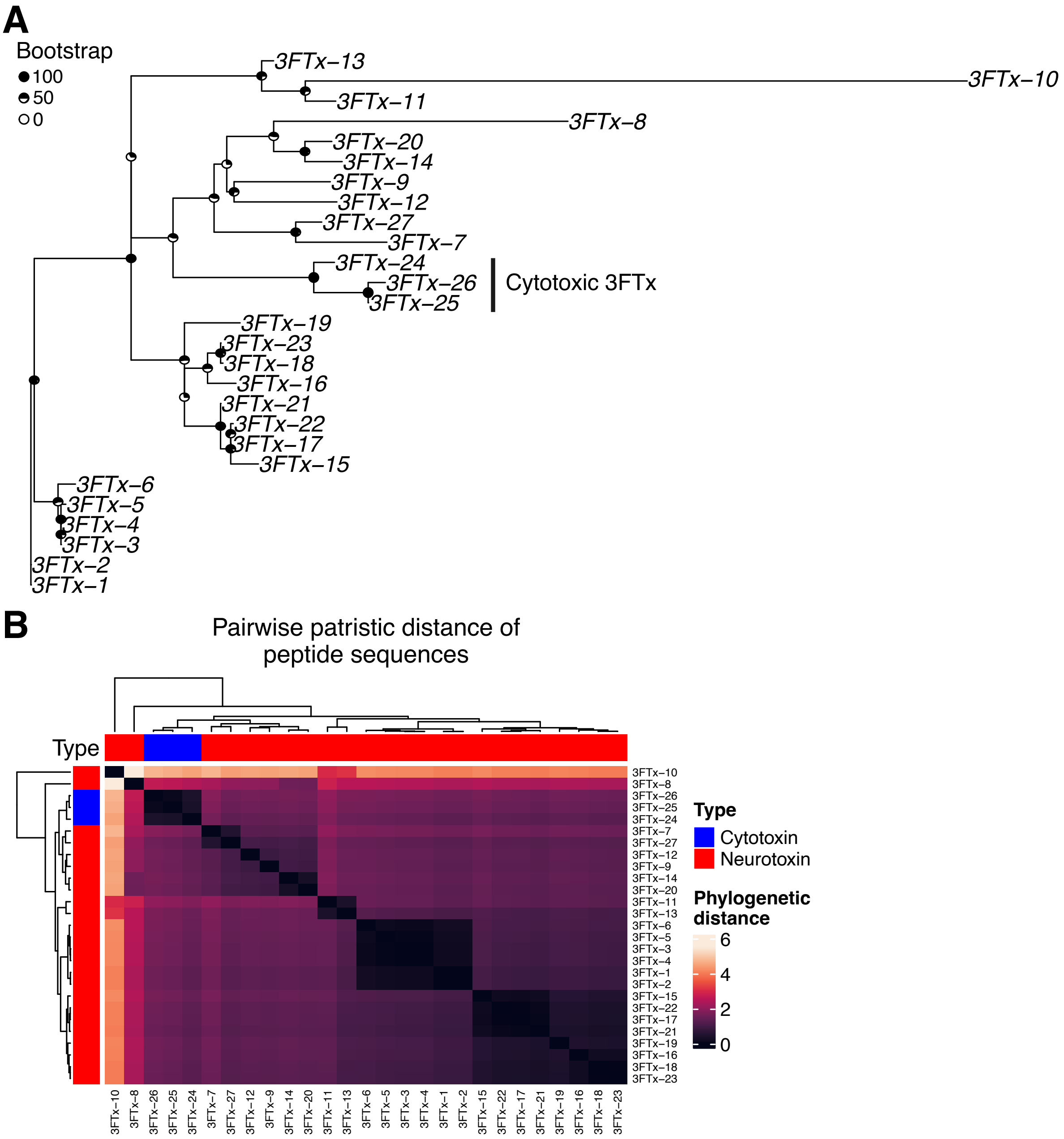

Figure S16: Phylogenetic relationships of peptide sequences of 3FTx genes. (A) Phylogenetic tree of peptide sequences. The support values of bootstrap are shown at tree nodes. This analysis revealed that the peptide sequence of the cytotoxic types of 3FTx are closely related when compared to the neurotoxic types. As previously observed using the mature peptide in Figure S3. (B) Pairwise phylogenetic (patristic) distance of 3FTx genes when analyzing their peptide sequences. Lighter colors indicate higher phylogenetic distance, whereas darker values indicate lower phylogenetic distance. The columns and row annotations indicate the neurotoxins (in red) or cytotoxins (in blue).

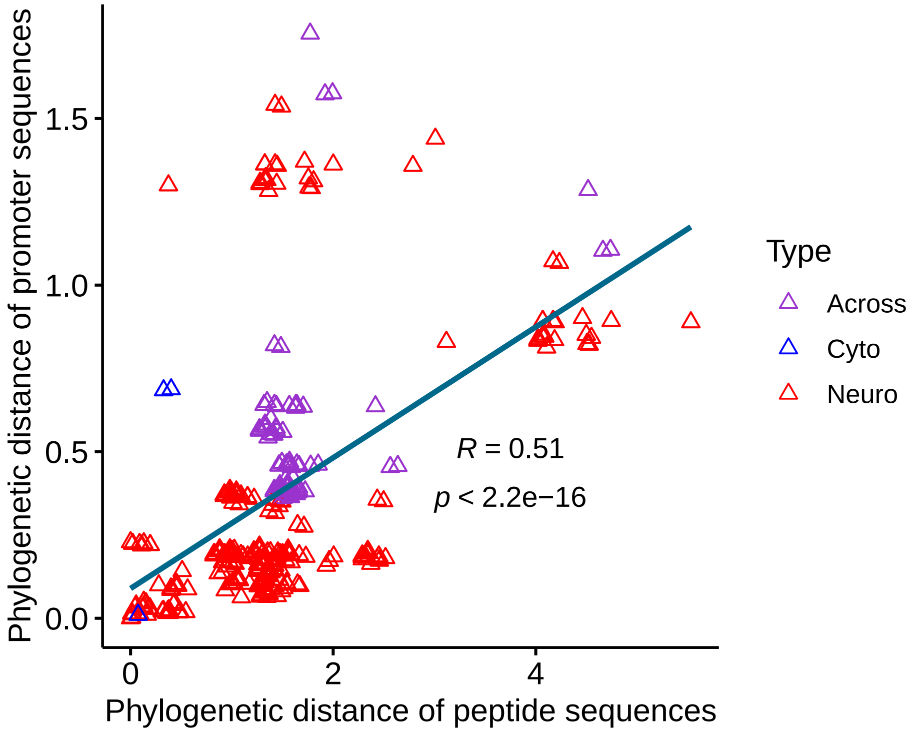

Figure S17: Correlation of peptide and promoter phylogenetic (patristic) distances of 3FTx genes. It follows the pairwise phylogenetic (patristic) distances calculated from gene trees in Figures S13 and S14. Comparisons within neurotoxins, within cytotoxins, and across them are coloured in red, blue, and purple, respectively. This analysis reveals a positive significant correlation of peptide and promoter sequences.

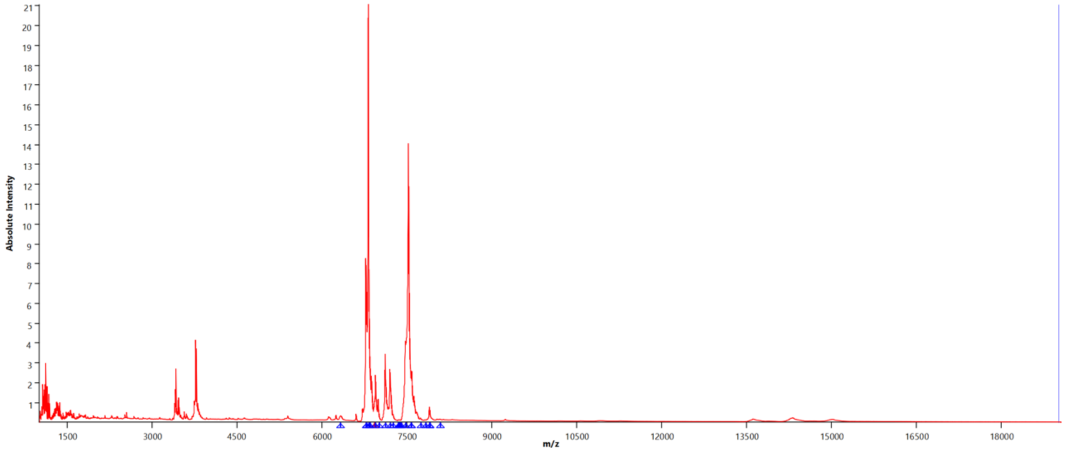

Figure S18: Average spectrum obtained in the mass spectrometry imaging using the venom gland section of *A. lubricus*. Blue triangles indicate m/z values of identified 3FTx. Peaks between 3000–4000 m/z are doubly charged 3FTx.

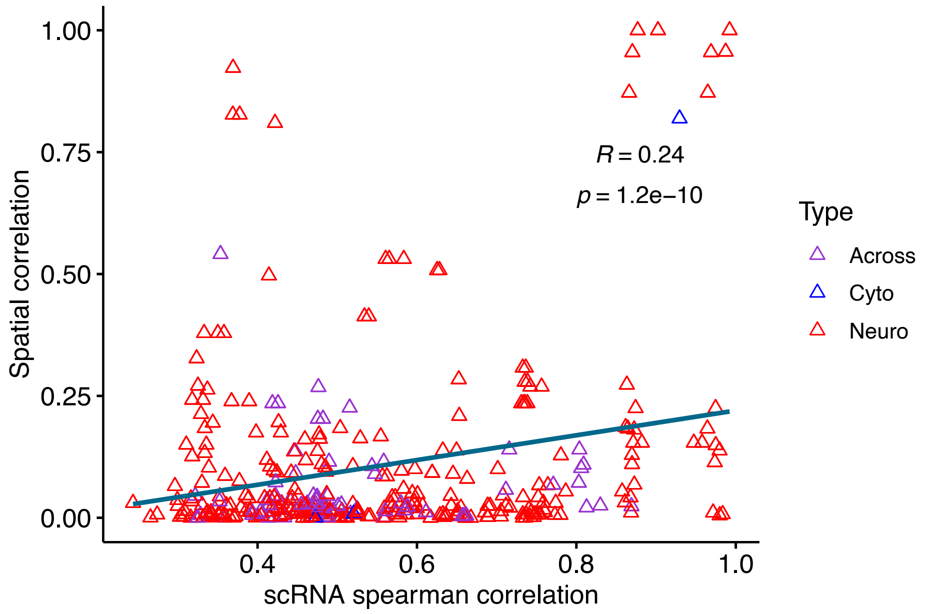

Figure S19: Correlation of spearman correlation of expression within cells in the scRNA-seq data (x-axis) and the spatial correlation of protein distribution within the venom gland (y-axis). This analysis suggests that heterogeneity is consistent at both transcriptomic and proteomic levels.

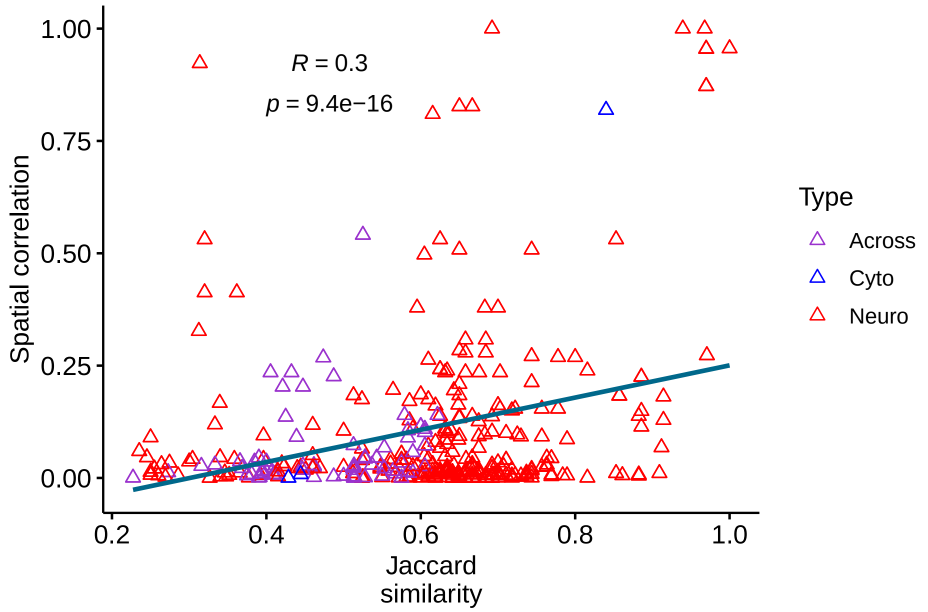

Figure S20: Correlation of Jaccard similarity of TFs using the 1,224 venom-gland cells (x-axis) and the spatial protein distribution within the venom gland (y-axis). This analysis suggests that modules of TFs may play major roles as regulators of the cellular heterogeneity observed in the *A. lubricus* venom gland.

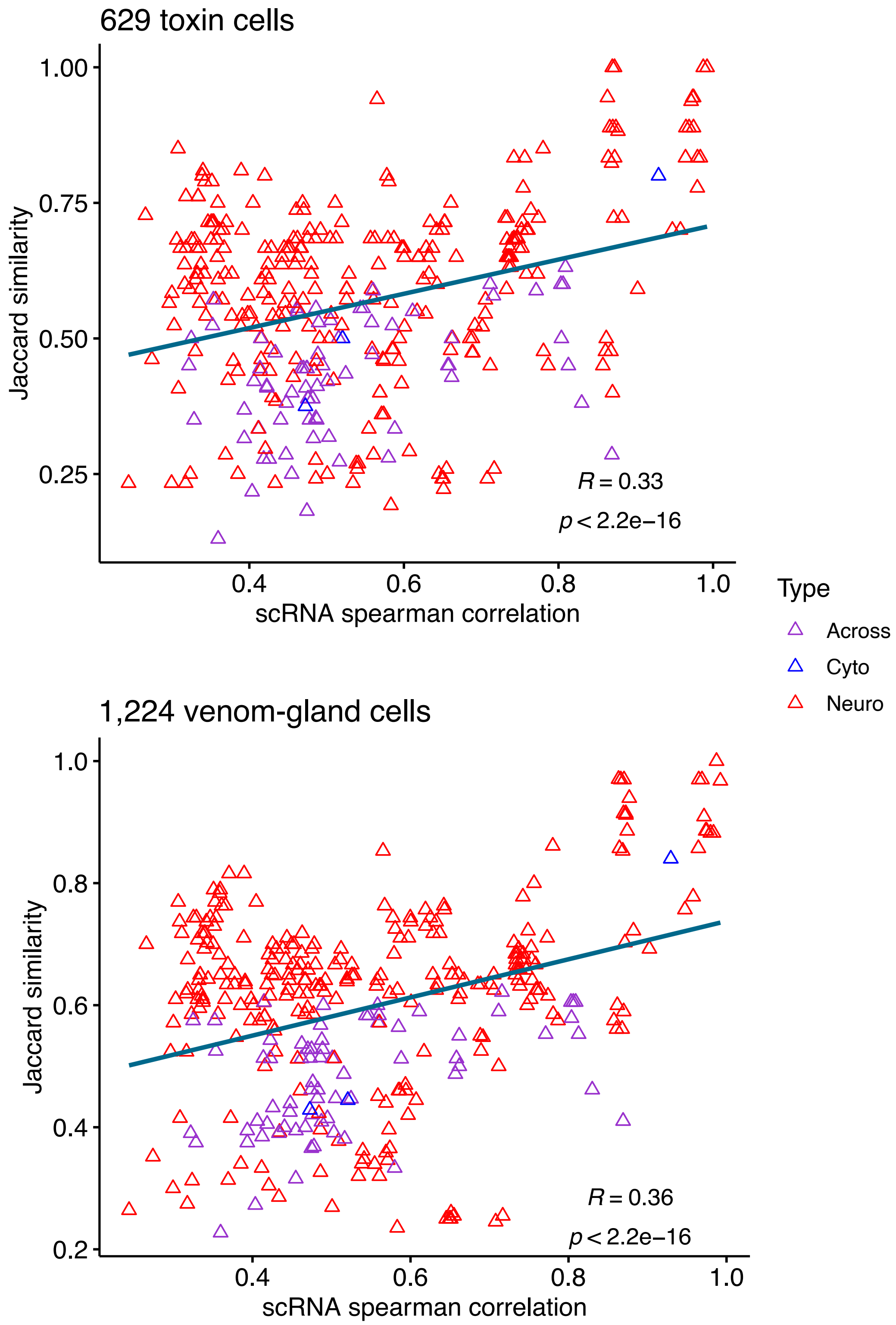

Figure S21: Correlation of spearman correlation of expression within cells in the scRNA-seq data (x-axis) and Jaccard similarity of TFs (y-axis) using the 629 toxin cells (at the top) and using the 1,224 venom-gland cells (at the bottom). This analysis suggests that modules of TFs may play major roles as regulators of the cellular heterogeneity observed in the *A. lubricus* venom gland.

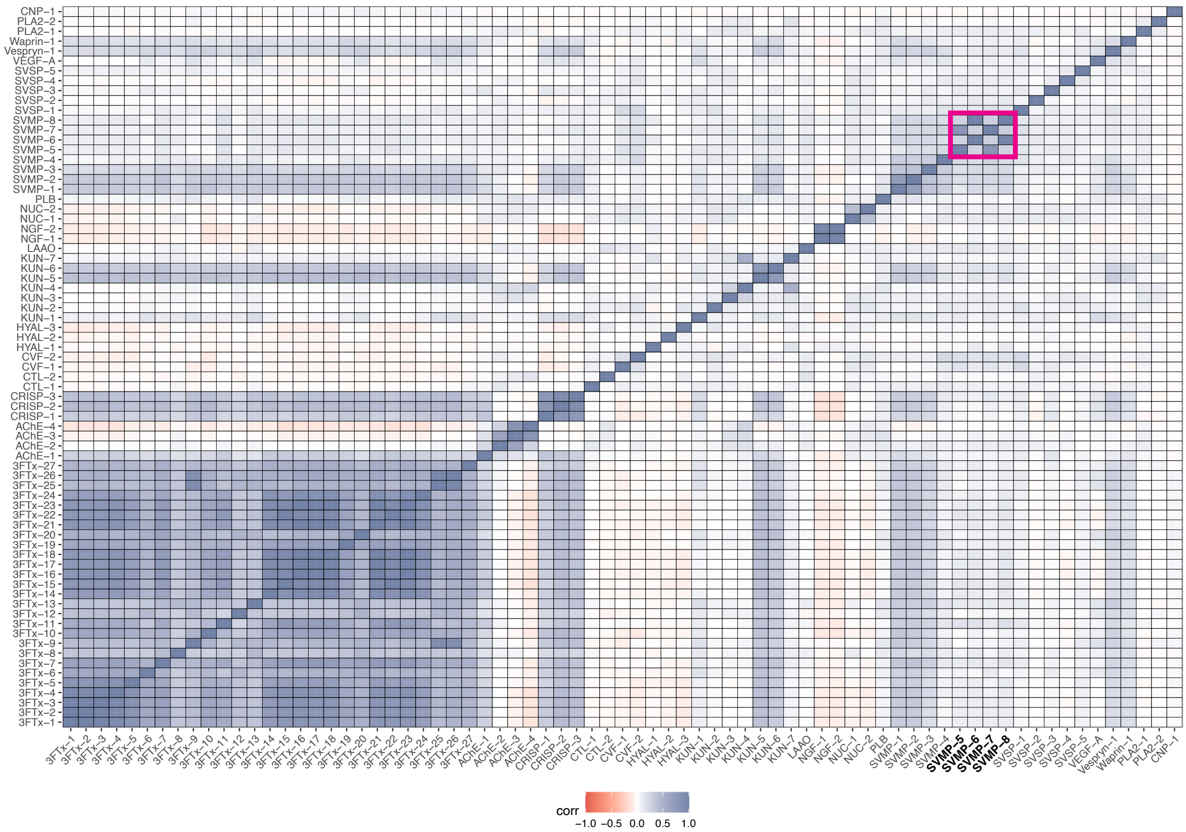

Figure S22: Spearman correlation of toxin expression within cells in the scRNA-seq data. The pink coloured box highlight the pairwise co-expression correlation of closely situated paralogous SVMP loci, which are also highlighted using bold font in the x-axis.

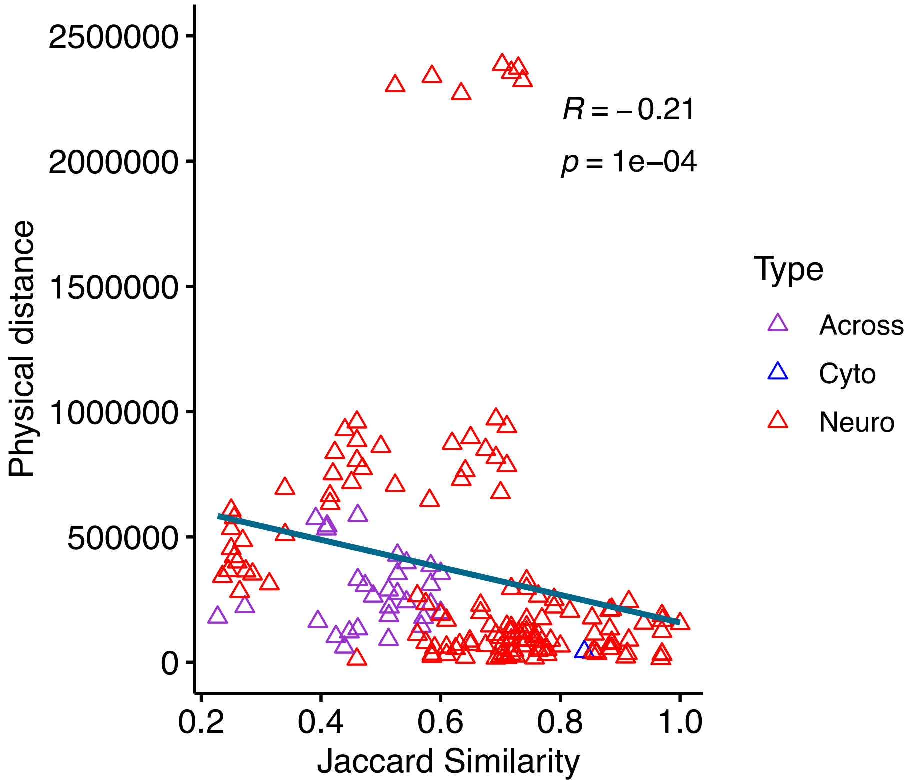

Figure S23: Correlation of Jaccard similarity of TFs (x-axis) and the physical genomic distance (y-axis) using the 1,224 venom-gland cells.

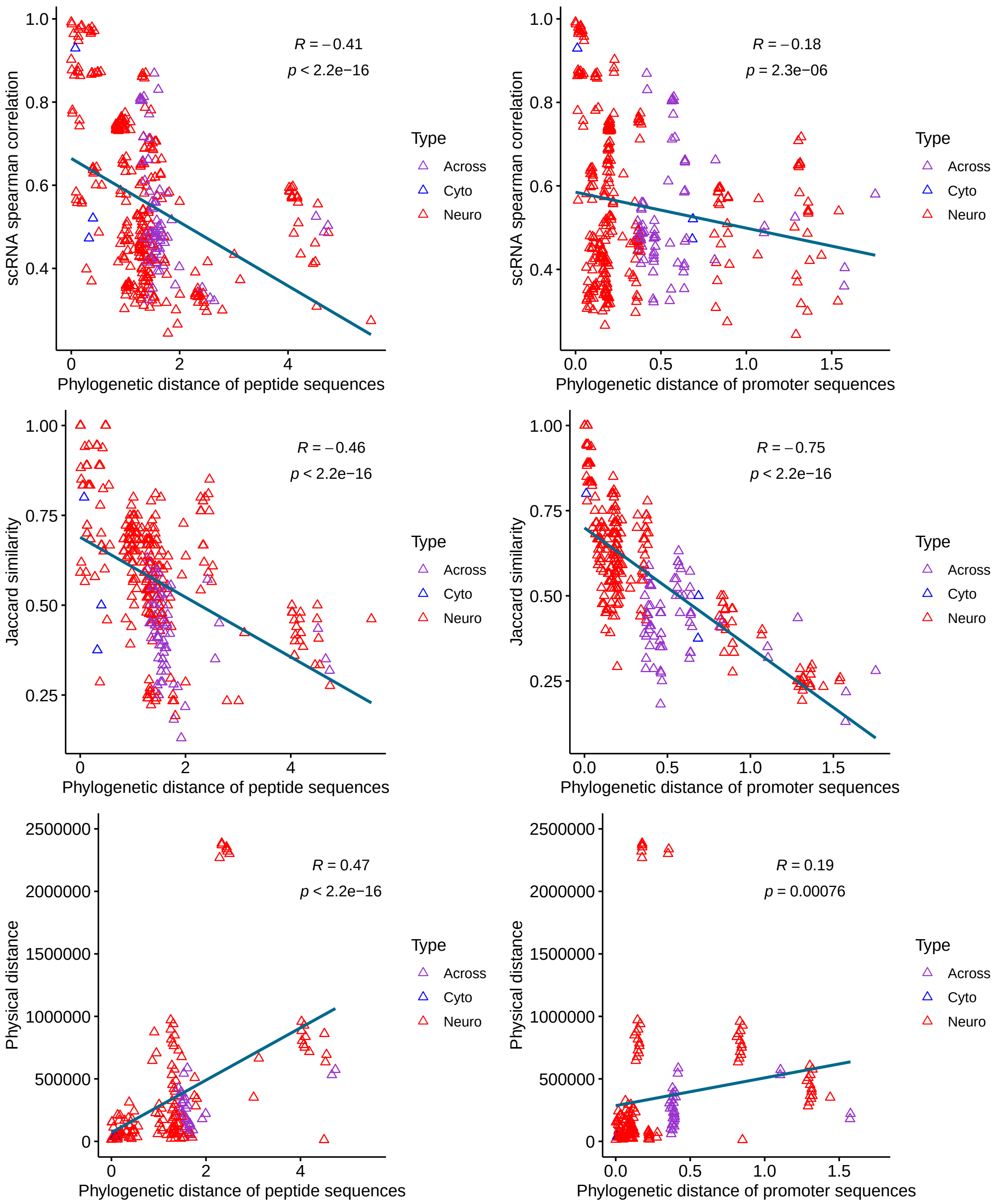

Figure S24: Correlation of phylogenetic distance of peptide (in the left) and promoter (in the right) sequences (x-axis) to the co-expression in scRNA data, Jaccard similarity of TFs, and the physical genomic distance (y-axis).

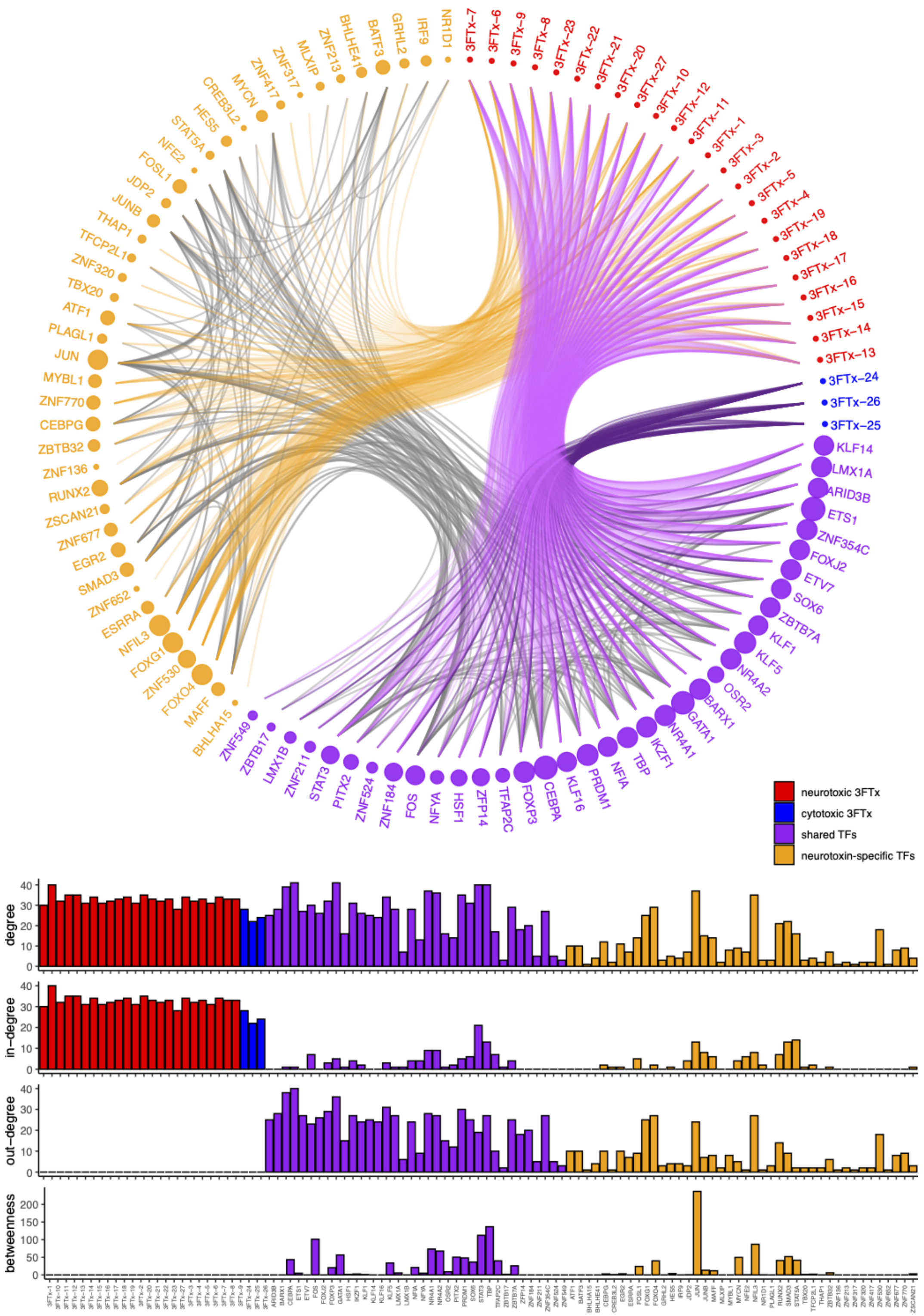

Figure S25: Genetic regulatory network (GRN) inferred for the cytotoxic and neurotoxic 3FTx using the 1,224 venom-gland cells. Neurotoxins, cytotoxins, shared TFs, and neurotoxin-specific TFs are coloured in red, blue, purple, and orange, respectively. The size of circles represents the out-degree of genes in the GRN. The orange edges indicate which neurotoxin-specific TF is binding to the neurotoxic 3FTx. The magenta and dark purple edges indicate which shared TF is binding to neurotoxins or cytotoxins, respectively. The gray edges indicate the protein-protein interactions between TFs retrieved from the STRING database. At the bottom, the degree, in-degree, out-degree, and betweenness centrality measures obtained for each gene in the GRN showing relevant TFs controlling the 3FTx expression profile observed.
